# Modelling the implications of stopping vector control for malaria control and elimination

**DOI:** 10.1186/s12936-017-2051-1

**Published:** 2017-10-13

**Authors:** Joshua O. Yukich, Nakul Chitnis

**Affiliations:** 1 Center for Applied Malaria Research and Evaluation, Tulane University School of Public Health and Tropical Medicine, 1440 Canal St. 8317, New Orleans, LA 70112 USA; 2 Swiss Tropical and Public Health Institute, Socinstrasse 57, 4051 Basel, Switzerland; 3 University of Basel, Basel, Switzerland

**Keywords:** Malaria, Elimination, Vector control resurgence

## Abstract

**Background:**

Increasing coverage of malaria vector control interventions globally has led to significant reductions in disease burden. However due to its high recurrent cost, there is a need to determine if and when vector control can be safely scaled back after transmission has been reduced.

**Methods and findings:**

A mathematical model of *Plasmodium falciparum* malaria epidemiology was simulated to determine the impact of scaling back vector control on transmission and disease. A regression analysis of simulation results was conducted to derive predicted probabilities of resurgence, severity of resurgence and time to resurgence under various settings. Results indicate that, in the absence of secular changes in transmission, there are few scenarios where vector control can be removed without high expectation of resurgence. These, potentially safe, scenarios are characterized by low historic entomological inoculation rates, successful vector control programmes that achieve elimination or near elimination, and effective surveillance systems with high coverage and effective treatment of malaria cases.

**Conclusions:**

Programmes and funding agencies considering scaling back or withdrawing vector control from previously malaria endemic areas need to first carefully consider current receptivity and other available interventions in a risk assessment. Surveillance for resurgence needs to be continuously conducted over a long period of time in order to ensure a rapid response should vector control be withdrawn.

**Electronic supplementary material:**

The online version of this article (doi:10.1186/s12936-017-2051-1) contains supplementary material, which is available to authorized users.

## Background

The World Health Organization (WHO) Global Malaria Programme’s policy of universal coverage of long-lasting insecticidal nets (LLINs) and/or indoor residual spraying (IRS) of people living in areas of malaria transmission, has led to large increases in global coverage of vector control and consequently to large decreases in malaria cases and deaths [1–3]. This increase in coverage, however, is expensive and many national malaria control programmes rely on international donors to support the financial costs of vector control implementation. With uncertainty over domestic and global financing, especially after malaria burden has been substantially reduced, control programmes may consider reducing funding for vector control. In this context it is critical to understand the probability of occurrence and potential severity of any resurgence of malaria, after a reduction in coverage of vector control interventions.

Few published studies and no randomized control trials have specifically investigated vector control withdrawal or reduction either before or after local elimination, although there is at least one randomized control trial currently underway, in South Africa (*Pers. com.* Immo Kleinschmidt). Cohen et al. conducted a systematic review of the published and grey literature to identify events of malaria resurgence [4], defining resurgence as “an increasing trend in malaria incidence or prevalence following suppression achieved through implementation of control efforts.” They identified 75 resurgence events in 61 countries from the 1930s through the 2000s, of which 91% involved weakening of the control programme, largely consisting of reduction in the level of vector control effort or outright vector control withdrawal. Reductions in funding was the most common specifically cited reason for the weakening of the control programme in question (49%). They state that:

*Reasons for funding reductions or cessation were not clear for all events, but in several, donors appear to have reallocated funding specifically* ***because*** *successful reductions in malaria burden had occurred.* [Emphasis ours]

Other recent studies in Zambia and Benin have also shown that withdrawal or relaxation of vector control efforts can lead, over short time periods, to resurgences in malaria prevalence, clinical incidence and transmission [5, 6]. The Cohen et al. review makes it clear that reductions in funding and weakening of control programmes, especially the withdrawal of vector control, are strongly associated with resurgence. However, it only examined events where resurgence *did* occur, and did not include cases where vector control interventions were withdrawn with no resurgence. A full picture of the risk of reductions in vector control effort or withdrawal must also consider the potential for resurgence not to occur after reductions in or withdrawal of vector control.

There is extensive experience with the scale back of vector control in near elimination or post local elimination settings; however only one published controlled study was found where withdrawal of vector control occurred in a setting in which elimination was not achieved and no resurgence was reported [7]. In that study, which was conducted in a low transmission area with highly zoophilic vectors, IRS with deltamethrin was delivered for approximately 3 years at high intensity and then withdrawn after the annual parasite index (API) fell by nearly 90% to below one per 10,000 per year, with follow-up studies conducted over a period of 10 years. Although the authors reported no resurgence, API and the slide positivity rate returned to levels comparable to an un-sprayed control area by the end of the study—but only after a period of nearly 10 years. (However, vector abundance measures returned to comparable levels to the control areas after only approximately 5  years).

In near elimination settings during the Global Malaria Eradication Programme (GMEP), transition from the “attack” (universal coverage with vector control interventions) to “consolidation” (withdrawal of universal vector control and reliance on surveillance and focal vector control) programme phases was advocated once a programme reached an incidence of infection consistent with local transmission interruption. Initially, the programme had determined a combined set of criteria of a local API less than 5 per 10,000 per annum and annual blood examination rate (ABER) greater than 10%, levels thought to be consistent with interruption of local transmission. These criteria were later revised to advocate for transition to the consolidation phase only when API fell to less than 1 per 10,000 per annum, because practical experience in the field suggested that the higher threshold resulted in substantial occurrences of resurgence, possibly due to bias in programme reporting of surveillance coverage [8–10]. These criteria may be appropriate in lower endemicity settings but have not been tested in higher endemicity African settings where the GMEP was largely absent.

A recent review of post malaria elimination transmission data indicated that in many countries which had successfully eliminated malaria, the risk of re-emergence of endemic malaria transmission was low and that the reproductive number for malaria in these locations had generally fallen dramatically post-elimination. This seems to have occurred despite the fact that much malaria vector control had not been indefinitely sustained [11]. While postulating that these changes might be caused by successful malaria elimination, the review also highlighted the fact that countries with successful elimination were different in many background characteristics than those countries which did not eliminate but only controlled malaria. Additionally, of the thirty countries with enough data to estimate reproductive numbers after elimination in this study, none were located in sub-Saharan Africa.

Whether or not vector control can be scaled back or withdrawn after local elimination (even sub-national elimination) largely depends on assessment of the risk of re-establishment of endemic malaria transmission or the malariogenic potential of the area [8, 9, 12]. This assessment in turn depends largely on whether significant importation risk (vulnerability) and transmission risk (receptivity) remain.

Additionally, the decision to safely scale back vector control in areas with historical malaria transmission and high coverage of vector control interventions requires the definition of a set of indicators which can specifically identify locations and times in which the scaling back of vector control might be safely undertaken. It further requires an understanding of the precision and bias associated with these measurements on estimates of the risk of resurgence following the scale back of vector control.

Although mathematical and simulation models of malaria have been used extensively to improve understanding of malaria transmission and to compare the effectiveness and cost-effectiveness of current and new interventions, they have focused on the impact of increasing the coverage of interventions and not on decreasing the coverage, as is considered here. This manuscript outlines the use of Monte Carlo simulation methods and mathematical models of *Plasmodium falciparum* malaria transmission (OpenMalaria) to address the above questions. Although simulations were only run for *P. falciparum* transmission, the implications for *Plasmodium vivax* transmission are considered in the Discussion.

## Methods

### Outline

OpenMalaria is an open source simulation platform, consisting of an ensemble of models of malaria in humans integrated with a model of malaria in mosquitoes, that allows the comparison of the effectiveness and cost-effectiveness of current and planned control interventions in various settings [13]. The ensemble of stochastic individual-based models for malaria in humans, using a discrete time step of 5 days, have been fit to multiple field data sets, and include detailed aspects of malaria dynamics, such as demography; acquired immunity and super-infection; variations in parasite densities and infectiousness to mosquitoes within humans; and the clinical effects of malaria [14–16]. The population-based discrete time model for malaria transmission in mosquitoes includes multiple mosquito species, heterogeneity in human hosts, nonhuman hosts, and seasonality [17, 18]. Additional details of the models can be found in the references above, or on the OpenMalaria website [13], which also provides a link for downloading software to simulate the model with the baseline parameterizations provided in the Additional files 1 and 2.

Simulations of these models determined the effects of scaling back from universal coverage of vector control interventions, specifically long-lasting insecticidal nets (LLINs). Simulations were run with multiple random seeds to include the effects of stochasticity; different model versions to include uncertainty in underlying model assumptions; and multiple parameterizations to allow for various assumptions of base (pre-intervention) transmission level, coverage of indoor vector control interventions, rate of imported infections, and coverage of case management and mass treatment interventions. The outputs of the simulations include the number of episodes of uncomplicated malaria, and the probability of resurgence following the scaling back of vector control.

Baseline parameterizations of an African setting (based on western Kenya) and a Western Pacific setting (based on the Solomon Islands) were created that described the bionomics of mosquito vectors and the seasonal profile of transmission for those locations. Numerical simulations were run for a population of 10,000 humans of these baseline scenarios, and of scenarios with different coverage levels of vector control and active case detection interventions and varying levels of pre-intervention transmission, imported infections, and case management coverage. A population size of 10,000 humans was selected because it is large enough to account for stochastic variation in clinical incidence.

**Fig. 1:**
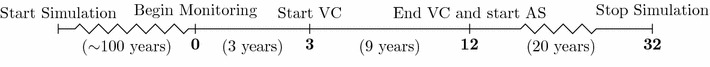
Schematic of generic scenario description for OpenMalaria simulations conducted for this study. “VC” stands for vector control and “AS” stands for active surveillance. Bold numbers indicate reference years for monitoring

The model was run for one human life span where humans are subjected to a periodically varying pre-intervention entomological inoculation rate (EIR) to induce malaria immunity in the human population and to estimate the mosquito emergence rate that leads to this EIR. After this warm-up period and a short stabilizing period, deployment of LLINs to humans occurs through four mass distribution campaigns, repeated every 3 years. Coinciding with the last deployment of nets, quarterly mass screen and treat campaigns simulate active case detection in the population for the remainder of the simulation. A schematic of the generic simulation scenario is shown in Fig. 1.

Monthly measurements of the annual EIR, the incidence of new infections—or force of infection (FOI), the number of patent infections, the number of uncomplicated clinical malaria cases per person per year, and the number of diagnostic tests were conducted for a total of 32 years. The first 3 years form the baseline period in the absence of any interventions (but with ongoing case management of clinical cases). The following 9 years form the vector control period (between the first and the fourth deployment of LLINs) where the probability of elimination is determined. The final 20 years form the post-vector control period where the probability of resurgence is determined. The post-vector control period begins directly after the final distribution of LLINs so a proportion of the population are initially protected by effective LLINs.

Since the simulations stochastically include imported infections, complete cessation of all transmission is unlikely. Therefore, elimination during the vector control period (between survey years *3* and *12*) is defined as occurring when the number of new infections in any 1 year is less than 3 times the 97.5 percentile of the Poisson distribution of the expected number of imported infections (in 1 year), as in a previous publication [19]. Similarly, resurgence in the post-vector control period (between survey years *12* and *32*) is defined as occurring when the number of new infections in any 1 year is greater than 3 times the 97.5 percentile of the Poisson distribution of the expected number of imported infections (in 1 year). In a sensitivity analysis, these thresholds are varied to between two and four times the importation rate to determine if the choice of threshold has a meaningful effect on the results. These definitions were chosen because: (A) The number of imported infections in a given simulation is Poisson distributed, thus the actual number of imported infections when accounting for stochasticity is 97.5% certain to fall below the 97.5 percentile of a Poisson distribution with mean and variance of the selected infection importation rate for that simulation. (B) If each imported infection produced two local cases, one would be certain that local transmission was reestablished. The definition of resurgence is independent of the definition of elimination, so resurgence could occur in a scenario regardless of whether or not elimination occurred in that scenario. Additionally, because the observation period post vector control was purposefully set to be longer than the vector control period, there is more opportunity to observe resurgence than elimination due to stochastic variation.

Time until resurgence is defined as the number of months before a monthly period where the number of new infections exceeded 3 times the 97.5 percentile of the Poisson distribution of the expected number of imported infections (in 1 month). As the time to resurgence analysis is conducted on a monthly scale, resurgence is more likely to occur in this analysis than in the definition of resurgence on an annual scale above.

Severity of resurgence in the post vector control period is defined using mean API with the equation,

$$\begin{aligned} \text {Severity of resurgence} = \overline{\text {API}}_{{\textit{12}-\textit{32}}} - \overline{\text {API}}_{{\textit{3}-\textit{12}}}, \end{aligned}$$

where $\overline{{API}}_{{\textit{3}-\textit{12}}}$ is the mean API during the vector control period and $\overline{\text {API}}_{{\textit{12}-\textit{32}}}$ is the mean API during the post-vector control period.

#### Baseline African scenario parameterization

The African scenario parameterization is based on a western Kenya setting from previously published work [20], including the transmission and entomological parameters (for *Anopheles gambiae*, *Anopheles funestus* and *Anopheles arabiensis*—except for seasonality profiles which were defined for the three vectors separately); the human demographic parameters; the parameterization of the initial effectiveness of LLINs and their rate of decay; the health systems parameters; and the profiles of the diagnostic tests and anti-malarial drugs used. This baseline parameterization can be found in the Additional file 1.

#### Baseline Western Pacific scenario parameterization

The Western Pacific scenario parameterization is based on the Solomon Islands, where the main vector species is *Anopheles farauti*. The seasonality profile for EIR is calculated from climate data using the EMOD model [21]. The extrinsic incubation period (10 days) and the duration of the mosquito resting period (2 days) are calculated from average temperature data in Guadalcanal using established relationships [22]. The value for the human blood index (72%) [23] and for the proportion of mosquitoes who are host-seeking on the same day that they laid eggs (73%) [24] are based on data for *An. farauti* from Papua New Guinea. The parous proportion of mosquitoes (58%) and the proportion of mosquitoes that bite indoors at times when humans are sleeping indoors (35%) are based on data from northern Guadalcanal [25]. The human demographic profile is estimated from United Nations population data [26]. Other parameters, such as the properties of the anti-malarial drugs and diagnostic tests, and the decay and effectiveness of LLINs are assumed to be similar to those used in the African scenario. This baseline parameterization can be found in the Additional file 2.

#### Experiment set-up

A full factorial design of varying the historical transmission level, the coverage of vector control interventions, the importation rate, the case management coverage, the intensity of active case detection, and the model assumptions, all for multiple random seeds to account for the effects of stochasticity, is performed to conduct a more thorough sensitivity analysis and simulate a wide range of potential settings.

- Transmission level: Levels of baseline (pre-intervention) EIR of {0.1, 0.5, 1, 2, 5} infectious bites per adult per year are simulated to represent a range of historical transmission intensities, with an upper limit of 5 infectious bites per adult per year because it is unlikely that an area with a higher historical transmission rate would consider withdrawing vector control unless it could permanently reduce receptivity.
- Coverage of vector control interventions: Coverage of LLINs is varied with values of {0, 0.2, 0.5, 0.8} of the proportion of the population sleeping under an LLIN on a given night during the VC period to simulate a wide range of vector control coverage.
- Importation rate: Importation rates of {0.1, 1, 10} infections per 1000 people per year are chosen to simulate a reasonable range of potential importation. Imported infections are simulated as new infections (not caused by the local mosquito population) in randomly chosen individuals with a probability drawn from a Poisson distribution, implicitly assuming that the imported cases have a similar level of acquired immunity to the local population.
- Case management coverage: Values of 20, 50, 80% are assumed for the percentage of all uncomplicated malaria cases are treated effectively to simulate a wide range of case management coverage.
- Active case detection: Mass screen and treat (MSAT) interventions every 3 months using rapid diagnostic tests (RDTs) and artemether–lumefantrine is simulated at coverage levels of {0, 2.5, 10, 20%} of the population to model increased active surveillance. Although it is unlikely that any programme would sustain quarterly MSAT campaigns of 20% of its population for 20  years, this is included as an approximation of targeted active surveillance.
- Model variants: Fourteen model variants, as described in more detail below, from a previous publication [16] are simulated to explore the implications of various model assumptions such as varying rates of decay of acquired immunity in humans and correlations of heterogeneities in humans.
- Stochasticity: Ten random seeds per model parameterization are simulated to include the effects of stochasticity.

### Model variants

OpenMalaria contains 14 different model variants so that uncertainty in model assumptions can be explored:

- R0001: Base OpenMalaria model.
- R0063: Mass action of the force of infection.
- R0065: Mass action of the force of infection.
- R0068: Mass action of the force of infection.
- R0111: Fixed decay in effective cumulative exposure (half-life 1000 years).
- R0115: Fixed decay in effective cumulative exposure (half-life 10 years).
- R0121: Fixed decay in immune proxies (half-life 1000 years).
- R0125: Fixed decay in immune proxies (half-life 10 years).
- R0131: Estimation of decay in effective cumulative exposure (half-life 1187 years).
- R0132: Estimation of decay in immune proxies (half-life 14 years).
- R0133: Estimation of decay in effective cumulative exposure (half-life 250 years) and immune proxies (half-life 19 years).
- R0670: Heterogeneity in susceptibility to comorbidity.
- R0674: Uncorrelated heterogeneities in access to treatment and susceptibility to comorbidity.
- R0678: Heterogeneity in access to treatment.

These variants and their parameterizations are described in more detail in a previous publication [16, Table 2, Text S1]. Some variants describe the same model with different parameterizations. Models R0111, R0115 and R0131 allow a decay in the memory of cumulative previous exposure to malaria (that leads to acquired immunity); models R0121, R0125 and R0132 allow a decay in the effectiveness of acquired immunity in reducing asexual parasite densities; and model R0133 allows both kinds of decays.

### Precision and bias

In order to examine the potential for surveillance systems to mis-measure or mis-classify important metrics suggested here as tools for determining the safety of vector control withdrawal, several additional simulation exercises using Monte Carlo simulation algorithms with the R software package [27] were conducted to estimate the precision and bias inherent in measurements of the infection importation rate (IIR) and ABER. Details of these simulation methods and results are included in the Additional file 3.

## Results

OpenMalaria simulations of stopping the deployment of vector control in an African setting are presented here; parallel results of simulations in a Western Pacific setting are presented in the Additional file 3. Results of the Monte Carlo simulations estimating the precision and bias inherent in measurements of the IIR and ABER are also presented in the Additional file 3.

### Descriptive results of OpenMalaria simulation outputs

**Fig. 2:**
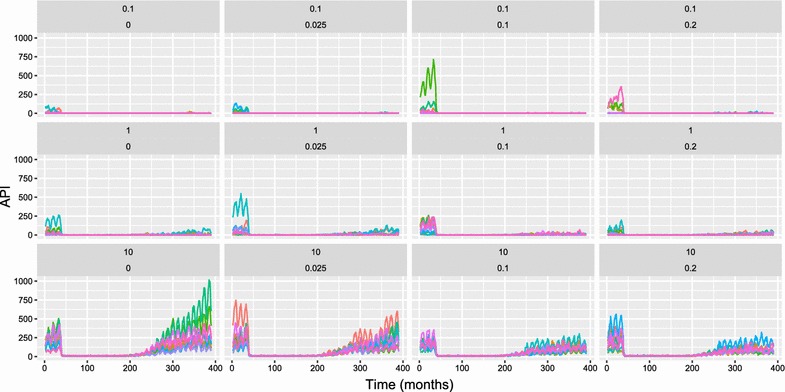
OpenMalaria simulation results for API per 1000 per annum (African scenario) with an annual pre-intervention EIR of 0.1 infectious bites per adult per year, case management coverage of 80%, and LLIN coverage of 80% during the period of vector control implementation. API is the annual parasite incidence computed at each time step and the x-axis is in months. Each chart shows simulations results for varied levels of IIR and active surveillance (through quarterly MSAT coverage). These values are shown just above each chart in the form: IIR per thousand per year (top line), proportion of population tested per quarter (second line). Colours of lines within the chart represent simulation runs with different random seeds (thus capturing stochastic uncertainty). LLINs are distributed at months 36, 72, 108, 144. Increased active surveillance starts immediately coincident with the last distribution of LLINs

**Fig. 3:**
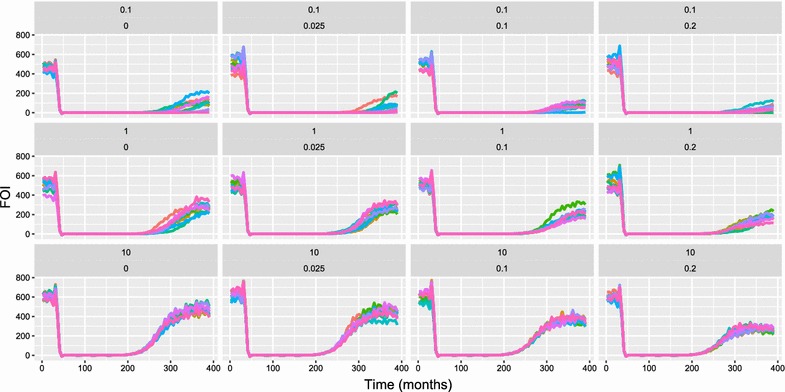
OpenMalaria simulation results for FOI per 1000 per annum (African scenario) with an annual pre-intervention EIR of 1 infectious bite per adult per year, case management coverage of 80%, and LLIN coverage of 80% during the period of vector control implementation. FOI is the number of new infections (including super-infections) over the previous month (normalised to units of infections per 1000 people per year) and the x-axis is in months. Each chart shows simulations results for varied levels of IIR and active surveillance (through quarterly MSAT coverage). These values are shown just above each chart in the form: IIR per thousand per year (top line), proportion of population tested per quarter (second line). Colours of lines within the chart represent simulation runs with different random seeds (thus capturing stochastic uncertainty). LLINs are distributed at months 36, 72, 108, 144. Increased active surveillance starts immediately coincident with the last distribution of LLINs

**Fig. 4:**
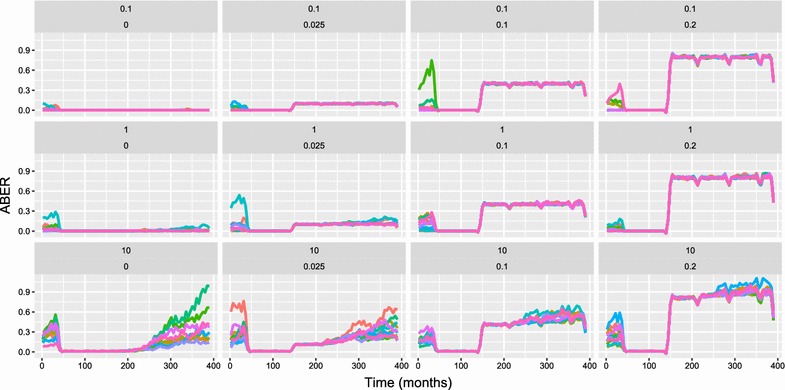
OpenMalaria simulation results for ABER (African scenario) with an annual pre-intervention EIR of 0.1 infectious bites per adult per year, case management coverage of 80%, and LLIN coverage of 80% during the period of vector control implementation. ABER represents the number of diagnostic tests used per person over the previous month (smoothed to remove the visual effects of widely varying ABER between time periods with quarterly MSAT surveys and normalised to units of tests per person per year) and the x-axis is in months. Each chart shows simulations results for varied levels of IIR and active surveillance (through quarterly MSAT coverage). These values are shown just above each chart in the form: IIR per thousand per year (top line), proportion of population tested per quarter (second line). Colours of lines within the chart represent simulation runs with different random seeds (thus capturing stochastic uncertainty). LLINs are distributed at months 36, 72, 108, 144. Increased active surveillance starts immediately coincident with the last distribution of LLINs

Simulation outputs allowed for the calculation of the time course of API, the force of infection (FOI) and ABER. Fig. 2 shows a sample of the simulation results for API with ongoing transmission in the baseline period in the first 3 years (where there are no interventions), little to no transmission during the vector control period, and resurgence in some of the simulations in the post-vector control period. API provides a metric, that is relatively easy to measure in the field, for estimating true infection incidence, especially at high case management coverage and low entomological inoculation rate (EIR). However, it can be biased by health system access (low case management coverage) and active surveillance activities, so it is not used here to define elimination and resurgence outcomes. Instead, these outcomes are defined using the number of new infections (including super-infections) at each model time step. Although FOI is sometimes defined as the number of new infections ignoring super-infections, and molecular FOI (or mFOI) defined as the number of new infections including super-infections, FOI is defined here as including super-infections. Indeed, at low values of EIR, in elimination and pre-elimination settings, super-infection is expected to be rare and the two metrics to be similar. Fig. 3 shows FOI for a subset of relevant simulation outputs, with similar patterns to the plots for API, but with resurgence apparent in most simulation runs. ABER provides a metric of the annual per capita number of diagnostic tests used. Results of a subset of simulations for ABER are shown in Fig. 4, where the number of tests used are dependent on transmission levels in the baseline and vector control periods, but increase due to active surveillance in the post vector control period.

Each simulation run was classified by whether elimination occurred or not (if transmission was interrupted by vector control) and whether or not resurgence occurred (if transmission was sustained after the deployment of vector control was stopped). Descriptive results are shown in Tables 1, 2, 3, 4, 5 and 6 for elimination and resurgence by various input parameters. Most simulations resulted in elimination during vector control roll-out. However, a smaller but similar fraction of simulations showed resurgence after vector control withdrawal (Table 1). When results for elimination and resurgence were examined in bivariate analysis for background characteristics of simulation, elimination during the vector-control period was associated with level of vector control coverage achieved (Table 2), case management coverage (Table 3), baseline pre-intervention EIR (Table 4), and model assumptions (Additional file 3: Table S1). Resurgence during the post-vector control period was associated with the level of vector control coverage achieved, case management coverage, baseline pre-intervention EIR, infection importation rate, active surveillance coverage (Tables 2, 3, 4, 5 and 6), and model assumptions (Additional file 3: Table S1).

**Table 1:** Simulation outputs for elimination and resurgence: here column labelled $\mathbf {Elim}$ shows the number of scenarios and the column labelled % shows the percentage of scenarios

Var.	Lev.	$\mathbf {Elim}_0$	%_0_	$\mathbf {Elim}_1$	%_1_	$\mathbf {Elim}_{\text{all}}$	%_all_
Elim.	0	31,232	100.0	0	0.0	31,232	31.0
	1	0	0.0	69,544	100.0	69,544	69.0
p < 0.0001	All	31,232	100.0	69,544	100.0	100,776	100.0
Resur.	0	1530	4.9	44,263	63.6	45,793	45.4
	1	29,702	95.1	25281	36.4	54,983	54.6
p < 0.0001	All	31,232	100.0	69,544	100.0	100,776	100.0

The subscript 0 denotes scenarios where elimination did not occur; 1 denotes scenarios where elimination occurred; and all summarizes the results for all scenarios. The rows for the variable, Elim. correspond to the scenarios where elimination occurred (Lev. 1) or did not occur (Lev. 0), and for the variable, Resur. correspond to the scenarios where resurgence occurred (Lev. 1) or did not occur (Lev. 0). The entries for the rows for elimination are trivial in this table but are included here for consistency with the other tables

Overall, there were 100,776 successfully completed simulations (a small number of simulation runs (24) failed to complete). Table 1 shows the proportion of simulations which resulted in elimination and/or resurgence. In the majority of simulations (69%) the level of malaria transmission during vector control deployment met the criteria for elimination during vector control deployment. The majority of simulations (55%) also resulted in a resurgence after withdrawal of vector control. Of the scenarios where malaria was eliminated, a majority showed no resurgence (64%). However, a substantial proportion (36%) showed resurgence after malaria had been eliminated. In a small proportion (5%) of scenarios, malaria was not eliminated but there was also no resurgence (due to stochastic variation and increased active surveillance in the scenarios where coverage of vector control was 0%). Since the definition of resurgence only depends on the ratio of incidence (of infections) to the importation rate, it does not require elimination to have occurred earlier.

Increasing coverage of LLINs during vector control deployment was associated with an increased probability of elimination and as well as a reduced probability of resurgence (Table 2). Changes in the level of case management coverage were associated with differences in the probability of elimination and resurgence (Table 3). Baseline pre-intervention EIR was strongly associated with probabilities of both elimination and resurgence. These associations showed trends in the expected directions with elimination much less likely to occur at higher input EIRs and resurgence much more likely to occur at higher baseline EIRs (Table 4). IIR was associated with the probability of resurgence but not with elimination (Table 5), implying that where vector control was sufficient to eliminate malaria, it could do so even in the presence of higher importation rates. Changes in active surveillance across the range tested was not related to the probability of elimination. Increasing active surveillance coverage was associated with a downward trend in the probability of resurgence. Since active surveillance was not deployed during the period of vector control in these simulations the lack of any association with elimination during vector control is expected (Table 6).

**Table 2:** Simulation outputs for elimination and resurgence in terms of LLIN coverage during vector control: here column labeled ITN shows the number of scenarios and the column labeled % shows the percentage of scenarios

Var.	Lev.	$\mathbf {ITN}_0$	%_0_	$\mathbf {ITN}_{0.2}$	%_0.2_	$\mathbf {ITN}_{0.5}$	%_0.5_	$\mathbf {ITN}_{0.8}$	%_0.8_	ITN_all_	%_all_
Elim.	0	23,729	94.2	7397	29.4	106	0.4	0	0.0	31,232	31.0
	1	1467	5.8	17,797	70.6	25,084	99.6	25,196	100.0	69,544	69.0
p < 0.0001	All	25,196	100.0	25,194	100.0	25,190	100.0	25,196	100.0	100,776	100.0
Resur.	0	1741	6.9	11770	46.7	15,382	61.1	16,900	67.1	45,793	45.4
	1	23,455	93.1	13,424	53.3	9808	38.9	8296	32.9	54,983	54.6
p < 0.0001	All	25,196	100.0	25,194	100.0	25,190	100.0	25,196	100.0	100,776	100.0

The subscript denotes the proportion of the population sleeping under LLINs during the vector control period. The rows for the variable, Elim. correspond to the scenarios where elimination occurred (Lev. 1) or did not occur (Lev. 0), and for the variable, Resur. correspond to the scenarios where resurgence occurred (Lev. 1) or did not occur (Lev. 0)

The model assumptions, as denoted by the variant of the OpenMalaria model used, were also associated with the probabilities of resurgence and elimination (See Additional file 3: Table S1). Although the decay of immunity did not substantially vary the probability of elimination over 20 years, model variants that included heterogeneity in access to treatment showed a substantially higher probability of resurgence. Lowering the threshold for elimination or resurgence substantially lowered the fraction of simulations in which elimination occured and increased the fraction in which resurgence occurred, while increasing these thresholds increased the probability of elimination and decreased the probability of resurgence. Details of sensitivity analysis are presented in the Additional file 3.

**Table 3:** Simulation outputs for elimination and resurgence in terms of case management coverage: here column labelled $\mathbf {CM}$ shows the number of scenarios and the column labelled % shows the percentage of scenarios

Var.	Lev.	$\mathbf {CM_{\mathrm {0.2}}}$	$\mathbf {\%_{\mathrm {0.2}}}$	$\mathbf {CM_{\mathrm {0.5}}}$	$\mathbf {\%_{\mathrm {0.5}}}$	$\mathbf {CM_{\mathrm {0.8}}}$	$\mathbf {\%_{\mathrm {0.8}}}$	$\mathbf {CM_{\mathrm {all}}}$	$\mathbf {\%_{\mathrm {all}}}$
Elim.	0	11,439	34.0	10,269	30.6	9524	28.4	31,232	31.0
	1	22,161	66.0	23,326	69.4	24,057	71.6	69,544	69.0
p < 0.0001	All	33,600	100.0	33,595	100.0	33,581	100.0	100,776	100.0
Resur.	0	11,646	34.7	15,770	46.9	18,377	54.7	45,793	45.4
	1	21,954	65.3	17,825	53.1	15,204	45.3	54,983	54.6
p < 0.0001	All	33,600	100.0	33,595	100.0	33,581	100.0	100,776	100.0

The subscript denotes the proportion of cases of malaria receiving effective treatment. The rows for the variable, Elim. correspond to the scenarios where elimination occurred (Lev. 1) or did not occur (Lev. 0), and for the variable, Resur. correspond to the scenarios where resurgence occurred (Lev. 1) or did not occur (Lev. 0)

### Regression results

In order to estimate the impact of various predictors on the probability, timing and severity of resurgence following scale back of vector control in a multivariate framework, logistic, Cox-proportional hazards and linear regression were applied using the input parameters and malaria outcomes during vector control of each simulation as predictors; and the occurrence, time until resurgence event and severity of resurgence post-withdrawal as the outcomes.

Table 7 summarizes the results for the probability of resurgence. These results indicate that most parameters which were significant in bivariate analysis retained important predictive value for the probability of a resurgence in multivariate analysis. Overall model results reinforce the importance of pre-intervention EIR, case management coverage, active surveillance coverage, infection importation rate and the level of control success during vector control deployment as major driving factors in predicting the probability of resurgence after withdrawal.

**Table 4:** Simulation outputs for elimination and resurgence in terms of baseline pre-intervention EIR: here column labelled $\mathbf {EIR}$ shows the number of scenarios and the column labelled % shows the percentage of scenarios

Var.	Lev.	$\mathbf {EIR_{\mathrm {0.1}}}$	$\mathbf {\%_{\mathrm {0.1}}}$	$\mathbf {EIR_{\mathrm {0.5}}}$	$\mathbf {\%_{\mathrm {0.5}}}$	$\mathbf {EIR_{\mathrm {1}}}$	$\mathbf {\%_{\mathrm {1}}}$	$\mathbf {EIR_{\mathrm {2}}}$	$\mathbf {\%_{\mathrm {2}}}$	$\mathbf {EIR_{\mathrm {5}}}$	$\mathbf {\%_{\mathrm {5}}}$	$\mathbf {EIR_{\mathrm {all}}}$	$\mathbf {\%_{\mathrm {all}}}$
Elim.	0	3785	18.8	4886	24.2	5249	26.0	7205	35.7	10107	50.1	31,232	31.0
	1	16,352	81.2	15,274	75.8	14910	74.0	12,955	64.3	10,053	49.9	69,544	69.0
p < 0.0001	All	20,137	100.0	20,160	100.0	20159	100.0	20,160	100.0	20,160	100.0	100,776	100.0
Resur.	0	15,652	77.7	12,268	60.9	9390	46.6	6393	31.7	2090	10.4	45,793	45.4
	1	4485	22.3	7892	39.1	10769	53.4	13,767	68.3	18,070	89.6	54,983	54.6
p < 0.0001	All	20,137	100.0	20,160	100.0	20,159	100.0	20,160	100.0	20,160	100.0	100,776	100.0

The subscript denotes the pre-intervention EIR in units of infectious bites per adult per year. The rows for the variable, Elim. correspond to the scenarios where elimination occurred (Lev. 1) or did not occur (Lev. 0), and for the variable, Resur. correspond to the scenarios where resurgence occurred (Lev. 1) or did not occur (Lev. 0)

**Fig. 5:**
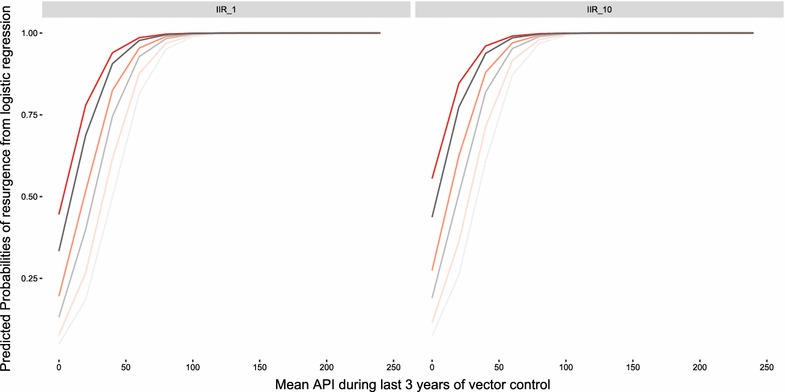
Predicted probabilities of resurgence based on logistic regression results plotted against the mean API during the last 3 years of vector control (years *9*–*12*). Darker lines represent increasing EIR (0.1, 1, 2) while red-orange lines represent active surveillance coverage of 1% per quarter and grey lines represent active surveillance coverage of 10% per quarter. The plot on the left is for an IIR of 1 imported infection per 1000 people per year while the plot on the right is for an IIR of 10 imported infections per 1000 people per year. All slopes here are for LLIN coverage of 80%, case management coverage of 50% and using the base model variant

These logistic regression model results can be used to summarize the predicted probability of a resurgence occurring with varying levels of input parameters. Fig. 5 shows the predicted probability of resurgence at varying levels of API, IIR, EIR and active surveillance coverage for the base model variant. The predicted probability of resurgence is generally high for most parameter combinations and only falls below 0.25 for a set of simulations in which pre-intervention EIR was less than 1 infectious bite per adult per year, IIR was 1 per 1000 per year, mean API during vector control deployment was below 25 per 1000 persons per year and there was some level of active surveillance. Although the definition of an acceptable probability of resurgence would need to context-specific, it is unlikely that a probability of resurgence greater than 0.25 would fall under this definition. Raising or lowering the threshold for elimination or resurgence did not substantially affect the magnitude or direction of any regression coefficients used for predicting the probability of resurgence. However, the changes produce noticeable shifts in the predicted probability of resurgence under different scenarios, indicating that understanding the background of transmission and the history of vector control in an area is important to determining the probability of resurgence. Further, these results show that the definition of resurgence is an important consideration in a full risk model. Full details of sensitivity analysis are presented in the Additional file 3.

The time to resurgence was analysed by fitting a Cox-proportional hazard model to these time to event outcomes, assuming that the simulations in which no resurgence occurred were right censored. The results of this regression are summarized in Table 8. All predictors for the probability of the occurrence of resurgence were similarly related to the time until resurgence, notably high vector control coverage, indicating strong suppression of malaria transmission during the vector control period was important not only to reducing the probability of resurgence occurring but also to delaying the occurrence of resurgence.

**Table 5:** Simulation outputs for elimination and resurgence in terms of infection importation rate per 1000 per annum: here column labelled $\mathbf {IIR}$ shows the number of scenarios and the column labelled % shows the percentage of scenarios

Var.	Lev.	$\mathbf {IIR_{\mathrm {0.1}}}$	$\mathbf {\%_{\mathrm {0.1}}}$	$\mathbf {IIR_{\mathrm {1}}}$	$\mathbf {\%_{\mathrm {1}}}$	$\mathbf {IIR_{\mathrm {10}}}$	$\mathbf {\%_{\mathrm {10}}}$	$\mathbf {IIR_{\mathrm {all}}}$	$\mathbf {\%_{\mathrm {all}}}$
Elim.	0	10,455	31.1	10,512	31.3	10,265	30.6	31,232	31.0
	1	23,134	68.9	23,083	68.7	23,327	69.4	69,544	69.0
p = 0.10	All	33,589	100.0	33,595	100.0	33,592	100.0	100,776	100.0
Resur.	0	21,085	62.8	14,281	42.5	10,427	31.0	45,793	45.4
	1	12,504	37.2	19,314	57.5	23,165	69.0	54,983	54.6
p < 0.0001	All	33,589	100.0	33,595	100.0	33,592	100.0	100,776	100.0

The subscript denotes the Infection Importation Rate per 1000 person-years. The rows for the variable, Elim. correspond to the scenarios where elimination occurred (Lev. 1) or did not occur (Lev. 0), and for the variable, Resur. correspond to the scenarios where resurgence occurred (Lev. 1) or did not occur (Lev. 0)

Linear regression to estimate the effects of the various parameters on severity of resurgence are presented in Table 9. Similarly, the main model parameters were all associated with severity in similar manners to the timing and probability outcomes. Higher LLIN coverage during the VC period was associated with less severe resurgences. Across all three outcomes, model variants when compared to the base model of malaria transmission dynamics [14] were associated with less severe resurgence, less likelihood of resurgence but shorter times to resurgence when it did occur.

**Table 6:** Simulation outputs for elimination and resurgence in terms of active surveillance coverage: here column labelled $\mathbf {AS}$ shows the number of scenarios and the column labelled % shows the percentage of scenarios

Var.	Lev.	$\mathbf {AS_{\mathrm {0}}}$	$\mathbf {\%_{\mathrm {0}}}$	$\mathbf {AS_{\mathrm {0.025}}}$	$\mathbf {\%_{\mathrm {0.025}}}$	$\mathbf {AS_{\mathrm {0.1}}}$	$\mathbf {\%_{\mathrm {0.1}}}$	$\mathbf {AS_{\mathrm {0.2}}}$	$\mathbf {\%_{\mathrm {0.2}}}$	$\mathbf {AS_{\mathrm {all}}}$	$\mathbf {\%_{\mathrm {all}}}$
Elim.	0	7804	31.0	7812	31.0	7830	31.1	7786	30.9	31,232	31.0
	1	17,389	69.0	17,382	69.0	17,367	68.9	17,406	69.1	69,544	69.0
p = 0.98	All	25,193	100.0	25,194	100.0	25,197	100.0	25,192	100.0	100,776	100.0
Resur.	0	10,499	41.7	10,793	42.8	11,672	46.3	12,829	50.9	45,793	45.4
	1	14,694	58.3	14,401	57.2	13,525	53.7	12,363	49.1	54,983	54.6
p < 0.0001	All	25,193	100.0	25,194	100.0	25,197	100.0	25,192	100.0	100,776	100.0

The subscript denotes the proportion of the population covered by active surveillance in each quarter. The rows for the variable, Elim. correspond to the scenarios where elimination occurred (Lev. 1) or did not occur (Lev. 0), and for the variable, Resur. correspond to the scenarios where resurgence occurred (Lev. 1) or did not occur (Lev. 0)

**Table 7:** Logistic regression of input model parameters on resurgence

Dependent variable:		
	Resurgence	95% C.I.
Mean API during VC (per 1000)	1.077***	(1.072, 1.082)
Case Management Cov.	0.021***	(0.019, 0.023)
EIR	3.304***	(3.239, 3.371)
(10x) Active Surv. Cov.	0.590***	(0.574, 0.606)
0.2 ITN	0.152***	(0.136, 0.170)
0.5 ITN	0.066***	(0.058, 0.075)
0.8 ITN	0.040***	(0.035, 0.045)
IIR 1	10.409***	(9.784, 11.079)
IIR 10	16.165***	(14.998, 17.427)
R0063	0.839***	(0.751, 0.938)
R0065	0.442***	(0.394, 0.496)
R0068	0.798***	(0.715, 0.892)
R0111	0.873**	(0.782, 0.975)
R0115	0.619***	(0.554, 0.692)
R0121	1.041	(0.933, 1.162)
R0125	1.356***	(1.217, 1.512)
R0131	1.344***	(1.205, 1.499)
R0132	1.860***	(1.669, 2.074)
R0133	1.241***	(1.113, 1.384)
R0670	1.068	(0.957, 1.192)
R0674	2.520***	(2.261, 2.810)
R0678	3.170***	(2.844, 3.535)
Constant	1.296***	(1.128, 1.488)
Observations	100,776	
Log Likelihood	− 27,995.100	
Akaike Inf. Crit.	56,036.200	

The number before “ITN” refers to the coverage of LLINs. The number after “IIR” refers to the importation rate in infections per 1000 people per year. The entries “R0063” through “R0678” refer to model variants (as described in more detail in the section, “Model variants”)

* p < 0.1

** p < 0.5

*** p < 0.01

## Discussion

Monte Carlo simulations were conducted to examine the precision and bias associated with IIR measurement and ABER measurement; and a full factorial simulation experiment using the OpenMalaria platform to identify determinants of potentially safe withdrawal of vector control. Overall, the results indicate that only in a minority of situations could withdrawal of vector control be expected to be safe (with a low probability of resurgence). These situations are characterized by low historic EIRs, low importation rates, highly successful vector control activities and high case management and surveillance coverage. In addition, ABER and the infection importation rate may be useful indicators for measuring importation risk (or vulnerability) and surveillance coverage. While both have significant potential for bias, in general the largest biases and the most important effects of their limited precision are likely to either result in conservative decisions, such as maintaining vector control, or to be of a small magnitude at relevant levels of the indicators. However, care should be taken to ensure that these indicators are measured in spatially (geographically) and temporally (seasonally) representative manners. Additionally, pre-intervention EIR remained strongly predictive after controlling for other inputs and programme measurable factors demonstrating that one of the most important independent predictors of malaria resurgence risk, timing and severity cannot be measured at the time the decision to withdraw vector control is made.

**Table 8:** Cox model regression of input model parameters on time to resurgence

Dependent variable		
	Time to resurgence	95% C.I.
Mean API during VC (per 1000)	1.038***	(1.036, 1.040)
Case Management Cov.	0.484***	(0.468, 0.500)
EIR	1.271***	(1.265, 1.278)
(10x) Active Surv. Cov.	0.901***	(0.893, 0.910)
ITN coverage	0.281***	(0.271, 0.291)
IIR	1.092***	(1.089, 1.095)
R0063	0.757***	(0.727, 0.788)
R0065	0.773***	(0.743, 0.804)
R0068	0.914***	(0.878, 0.951)
R0111	0.975	(0.936, 1.014)
R0115	0.958**	(0.921, 0.997)
R0121	1.022	(0.982, 1.063)
R0125	1.078***	(1.036, 1.121)
R0131	1.050**	(1.009, 1.092)
R0132	1.085***	(1.043, 1.129)
R0133	1.029	(0.989, 1.070)
R0670	1.032	(0.992, 1.073)
R0674	1.109***	(1.066, 1.154)
R0678	1.186***	(1.140, 1.234)
Observations	70,908	
R^2^	0.302	
Max. possible R^2^	1.000	
Log likelihood	− 694,204.000	
Wald test	26,609.350*** (df = 19)	
LR Test	25,491.790*** (df = 19)	
Score (Logrank) test	28,846.640*** (df = 19)	

The entries “R0063” through “R0678” refer to model variants (as described in more detail in the section, “Model variants”)

* p < 0.1

** p < 0.5

*** p < 0.01

**Table 9:** Linear regression of input model parameters on severity of resurgence

Dependent variable		
	Severity	
Case Management Coverage	− 22.556***	(− 24.298, − 20.813)
EIR	25.260***	(25.017, 25.502)
(10x) Active Surv. Cov.	− 22.729***	(− 23.277, − 22.180)
0.2 ITN	114.306***	(113.099, 115.513)
0.5 ITN	92.247***	(91.040, 93.454)
0.8 ITN	82.173***	(80.966, 83.380)
IIR 1	13.928***	(12.883, 14.973)
IIR 10	42.540***	(41.495, 43.586)
R0063	− 6.713***	(− 8.973, − 4.454)
R0065	− 9.736***	(− 11.994, − 7.477)
R0068	− 13.044***	(− 15.303, − 10.786)
R0111	− 0.193	(−2.451, 2.066)
R0115	− 3.134***	(− 5.392, − 0.876)
R0121	1.154	(− 1.104, 3.412)
R0125	7.293***	(5.035, 9.552)
R0131	3.567***	(1.309, 5.825)
R0132	9.814***	(7.556, 12.072)
R0133	4.119***	(1.861, 6.377)
R0670	2.113*	(− 0.145, 4.371)
R0674	18.419***	(16.161, 20.677)
R0678	19.057***	(16.799, 21.315)
Constant	− 84.218***	(− 86.361, − 82.076)
Observations	100,776	
R^2^	0.490	
Adjusted R^2^	0.490	
Residual Std. Error	69.120 (df = 100,754)	
F Statistic	4,603.479*** (df = 21; 100,754)	

The number before “ITN” refers to the coverage of LLINs. The number after “IIR” refers to the importation rate in infections per 1000 people per year. The entries “R0063” through “R0678” refer to model variants (as described in more detail in the section, “Model variants”)

* p < 0.1

** p < 0.5

*** p < 0.01

During the Global Malaria Eradication Programme (GMEP), many countries targeted specific regions for complete coverage with IRS. After the end of GMEP when vector control was withdrawn, some countries, such as Greece, Italy and the United States of America, remained malaria-free, as certified by the WHO. However, vector control is still sometimes deployed in these countries and/or often remains a part of a response strategy to introduced malaria cases (often focally around the cases), and malaria transmission potential may remain indefinitely [28]—as recent outbreaks of autochthonous transmission in the Bahamas, Greece, Singapore and the United States of America, demonstrate [29–32]. Furthermore, and more crucially, many other countries saw large resurgences of malaria after vector control was withdrawn [4].

This confirms the expectation that return to an endemic equilibrium after withdrawal of vector control interventions may be slow in low transmission settings, but that it will eventually occur [33]. It also indicates that measurements of entomological and parasitological parameters shortly after the withdrawal of vector control will not be useful indicators of the propensity of an area to eventually re-equilibrate to historic transmission and disease levels.

Areas where vector control interventions had little to no impact on malaria transmission due to improper deployment, poor intervention choice or possibly resistance in the vector, may withdraw these interventions without much danger. However, given the broad utility of malaria vector control, especially the continued effectiveness of LLINs in the presence of pyrethroid resistance [34] and of adult vector control even with partially zoophagic and exophilic vectors [35, 36], the situations in which well deployed adult vector control has no meaningful impact on malaria transmission are expected to be limited, and such areas should focus on improving coverage of locally appropriate vector control interventions.

For areas that satisfy the general characteristics suggested to be safe for vector control withdrawal by this analysis, decisions to stop universal coverage of vector control interventions will need to be supported by further analyses that include more detailed descriptions of those settings to provide better calibrated risks. Additionally geo-spatial Bayesian analysis, if properly validated, can guide countries in safely transitioning from universal vector control to geographically targeted vector control, such as recent analysis of malaria receptivity in Somalia [37] and residual malaria transmission in Swaziland [38].

### Limitations

This study relies on Monte Carlo simulation and stochastic individual-based simulation models of malaria epidemiology and immunology. While mathematical modelling techniques have been useful in understanding malaria epidemiology and planning control, they contain inherent simplifications of the real world [39]. Model structures and assumptions can result in biases inherent in the models and limit their use for predicting real world outcomes.

This analysis, using OpenMalaria, mainly considered the effects of changes in immunity and increased active case detection in preventing resurgence. Active case detection was modelled as repeated regular rounds of mass test and treat at various levels of coverage. This serves as a crude approximation to the increased surveillance and potential reactive case detection that could be deployed to prevent resurgence. Reactive case detection is a form of test and treat that may have an increased likelihood of finding cases (that varies with prevalence), than mass test and treat. This model captures the increased likelihood of reactive case management—by assuming a higher coverage—but does not capture the dependence on prevalence. Other focal strategies such the spatial targeting of interventions around index cases (including focal vector control) or control based on other local circumstantial knowledge were also not considered. Such strategies are likely to be an important facet of scaling back from universal coverage of vector control interventions in some situations and the results in this manuscript do not explicitly capture this possibility.

In these simulations, receptivity of an area was assumed to be stable. This excludes the potential effects of secular changes such as improved housing and general economic development on the likelihood of resurgence. These changes are likely to occur over the 20 year post-vector control period, but quantifying the possible decrease in transmission potential is difficult and the focus here is only on those factors directly related to malaria transmission. Smith et al. [11] show that in addition to secular changes, decreasing malaria transmission may make resurgence more unlikely, for example due to decreasing immunity leading to a reduced probability of asymptomatic cases; and some of these effects are captured through OpenMalaria model variants that include the decay of immunity. Indeed, these model variants showed lower probabilities of resurgence and reduced severity of resurgence, suggesting that these effects are important, even though they also predicted shorter times until resurgence occurred.

Furthermore, the human malaria models of OpenMalaria have been developed and parameterized for *P. falciparum*. The effects of persistence in the liver stage of *P. vivax* would alter the dynamics of resurgence, so this would need to be considered for settings close to elimination where *P. vivax* predominates, or at least forms a substantial proportion of infections.

Imported infections in OpenMalaria are simulated by stochastically introducing an infection in a member of the local population. This corresponds to assuming that all imported infections arise from residents travelling abroad and returning with malaria. In reality, imported infections may also occur due to visitors (or immigrants) with substantially different immune profiles to those of the local population. Since areas considering scaling back vector control are likely to have little malaria, residents are likely to have low immunity to malaria. Therefore, visitors with different immune profiles to the local population would have high immunity to malaria and are likely to be asymptomatic. Short-term visitors could infect mosquitoes that lead to new infections in the local population (as is modelled here). Longer term visitors could remain a source of infection to mosquitoes until they self-cure or get clinically ill and seek treatment. This would be equivalent to a higher rate of secondary infections in the native population than is modelled here.

The analysis of the simulation results presented here depends on a particular definition of elimination and resurgence. This definition was chosen because it is consistent with previous work [19], and because it is strict and consistent with re-establishment of endemic transmission. Although other definitions may produce different conclusions, a sensitivity analysis of varying the threshold level showed little difference in the qualitative results. The proportion of scenarios showing elimination increased as the threshold was increased; and the proportion of scenarios showing resurgence decreased as the threshold was increased. However, the decrease in the proportion of scenarios showing resurgence was small, and the increase in the proportion of scenarios showing elimination was minimal. A consequence of using a definition based on IIR is that higher IIR scenarios can experience significantly more cases without being defined as resurgent. Another aspect of the definition is that it is limited to a defined temporal period. It is possible that the simulations that did not show resurgence would have shown resurgence if they had been conducted for a longer time span. However, this is likely mitigated by the long length of monitoring (20 years) after the withdrawal of vector control in these simulations.

The results of the statistical model emulation used to summarize the results of the simulation models are presented here with tests of statistical significance and associated *p*-values as is custom for standard statistical analysis and modelling. As the sample size in these model simulation runs is artificially constructed by the researchers the results of tests of statistical significance are somewhat irrelevant, and rather the direction of effect is more important. For this reason, the statistical significance of the results is not extensively discussed here. However, since it is possible for associations due purely to chance to arise because of the stochasticity inherent in these model formulations, the tests of significance are included here to help the reader discern which associations are likely true relationships present in the models and which are likely to be spurious or only weak associations.

## Conclusions

While these simulation results suggest that there are a set of scenarios in which it is possible to withdraw vector control without a significant probability of resurgence, they suggest that these situations are limited. Furthermore, there is no guarantee that resurgence will not occur even when its probability is low. Therefore, it is crucial that programmes maintain surveillance coverage (both clinical as well as entomological) not only for the benefits related to preventing resurgence, but also so that malaria control and elimination programmes which choose to scale back vector control are aware and prepared to make rapid responses should resurgence occur.

In areas with ongoing local malaria transmission, the scale-back from universal coverage of vector control is likely to lead to resurgence and a return to pre-intervention levels of malaria parasite transmission and disease. The speed and severity of such a resurgence might be exacerbated by high pre-intervention malaria transmission, poor vector control coverage before scaling back, and low case management coverage.

In areas in which local malaria transmission has been substantially reduced or interrupted, the scale-back of vector control is also associated with a high probability of resurgence for the vast majority of situations. The conditions which hold a low probability of resurgence include having a low pre-intervention EIR, high case management coverage, low importation rate of infections, and very successful control of transmission during the intervention period. The degree to which programmes can safely plan to withdraw or scale back vector control must be determined by the tolerance of a programme for risk of resurgence and its expected severity. When tolerance for the risk of resurgence is low, few situations would be *a priori* suitable for vector control withdrawal. If a 20% probability of resurgence is considered to be a threshold for safety, only scenarios with a pre-intervention EIR below 1 and moderate case management coverage (> 50%) with successful achievement of universal vector control coverage (> 80%) during the intervention phase were considered safe for withdrawal. These results held for both African and Western Pacific scenarios.

## Additional files

**Additional file 1.** Baseline parameterization of the African scenario.

**Additional file 2.** Baseline parameterization of the Western Pacific scenario.

**Additional file 3.** Supplemental simulation results and results of precision and bias simulations.
