# Supplementary material for: Modelling the implications of stopping vector control for malaria control and elimination
Source: Malar J. 2017 Oct 13;16:411. doi: 10.1186/s12936-017-2051-1 (PMC5640964; doi:10.1186/s12936-017-2051-1)
Supplement: Supplementary file 3 — Additional file 3. Supplemental simulation results and results of precision and bias simulations. [file 12936_2017_2051_MOESM3_ESM.pdf]

Modelling the implications of stopping vector control for malaria control and elimination

S1 Appendix

Joshua Yukich and Nakul Chitnis

17 August 2017

1 Precision and Bias Methods

1.1 Infection Importation Rate (IIR)

In order to estimate the precision and bias associated with measurement of IIR we conducted simulations of importation and measurement assuming that the number of importations weekly was given by a Poisson distribution with a mean of the true IIR. We then assumed that there was a observation process given by a binomial distribution which determined whether each of the imported infections was actually detected. We simulated this process for a one year period (52 weeks) and repeated the simulations for 10,000 iterations assuming varied mean true IIRs (from 1 per 1,000 persons per annum to 5 per 1,000 persons per annum) and varied detection rates (from 20% to 80%). We then tested each result against a threshold of 2 per 1,000 per annum to determine if, for each simulation, a Poisson significance test would determine that the number of imported infections per year would be determined to be statistically significantly below the threshold with $\geqslant 90\%$ confidence. This sequence of results were then analyzed with logistic regression and the predicted probability of concluding that IIR (based on the measurement) was below the threshold was summarised by true IIR and the detection probability in the surveillance system.

1.2 Annual Blood Examination Rate (ABER)

In order to estimate the potential bias associated with utilising ABER as a metric for surveillance system coverage we conducted simulations designed to determine the divergence between ABER and the total proportion of a population tested during one year with multiple active case searches covering varying proportions of the population where individuals have varied probabilities to be covered: in other words, where the active searches are likely to repeatedly test or miss the same individuals. We simulated a cohort of individuals with either independent probabilities of being

tested in each round, equal to the total proportion covered during said round, or by assuming that all individuals in the cohort had a constant predetermined probability of inclusion during all rounds. These probabilities were generated by simulating from a beta distribution with a known mean. The actual inclusion of an individual as tested in a round was drawn from a binomial distribution with probability determined in one of the two above methods. The annual blood examination rate was calculated as,

$$\text{ABER} = \frac{\text{Number of Tests Conducted}}{\text{Person-Years}}, \tag{1}$$

while the the proportion of the population actually tested was calculated as,

$$\text{PT} = \frac{\text{Number of Individuals Tested}}{\text{Person-Years}}. \tag{2}$$

2 Precision and Bias Results

The predicted probabilities from the logistic model for decisions based on IIR are shown in Figure S1. These results indicate that as the surveillance system improves (increases the probability of detecting imported infections) that there is relatively little chance of incorrectly concluding that the importation rate is below a specified threshold in error. However, the results also show that when the surveillance system has a high probability of detecting imported infections, programs will often not be able to conclude that the IIR is low enough to withdraw vector control unless the true IIR is significantly below the acceptable threshold of risk.

The results of simulation of ABER are shown in Figure S2. They indicate that although ABER and the proportion of the population actually tested by a surveillance system are likely to greatly diverge at high values, at the lower levels of interest here, they are likely to be largely similar. Thus at least at lower levels of testing, ABER is likely to be a reliable metric for the monitoring surveillance system coverage.

Results of an analysis of OpenMalaria simulation outputs indicates a further complication in monitoring and determining whether an area meets the acceptability threshold for withdraw of vector control, which is that the annual parasite index (API) and ABER are both highly correlated in these individual simulation outputs. This is likely because API is essentially a product of the positivity rate among those tested and ABER, therefore, API tends to increase with increases in ABER.

3 Supplemental Simulation Results

3.1 African Setting

Model variant was also significantly associated with the probability of resurgence and elimination (Table S1).

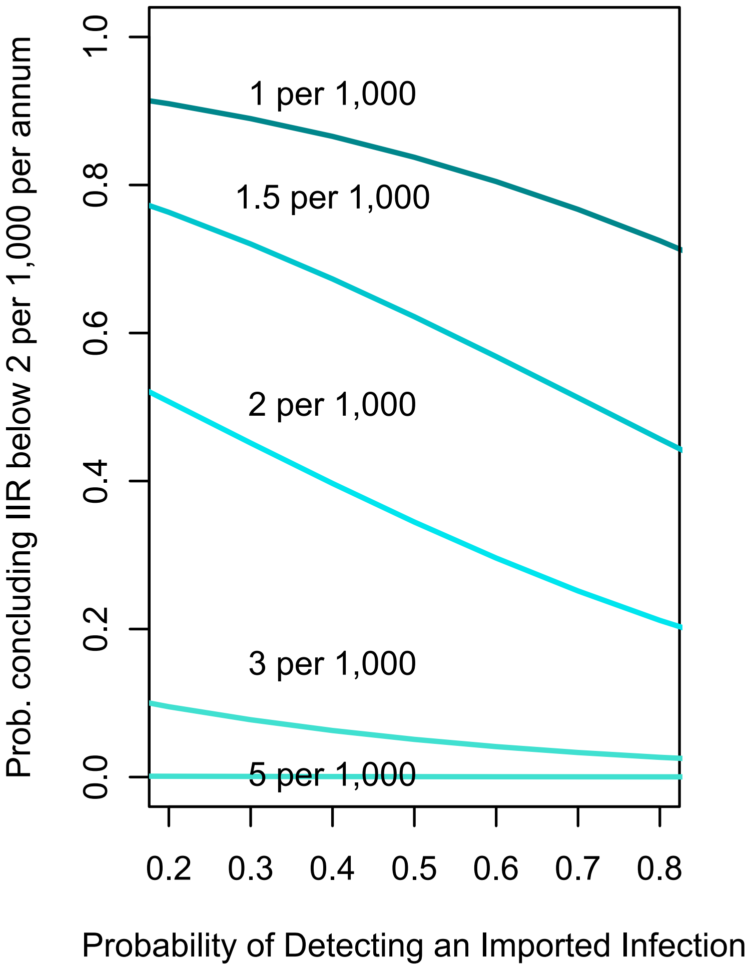

Figure S1: Simulation results for measurement of infection importation rate

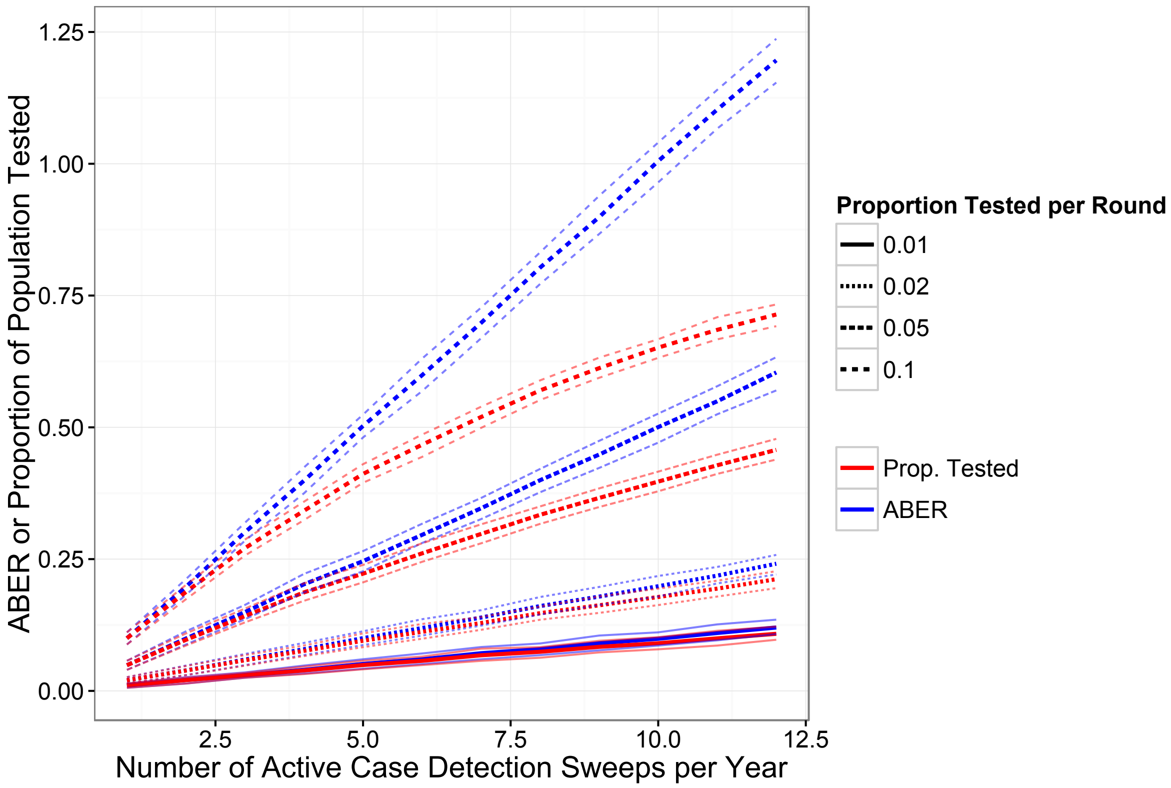

Figure S2: Simulation results for measurement of the annual blood examination rate. Here the detection probabilities for each individual are fixed and some individuals are more likely to be repeatedly tested. Differences between ABER and the true proportion of the population tested are more exaggerated in this model than in a random testing model. Small dotted lines give the bounds surrounding 95% of simulation results.

**Variable**	**Levels**	$\textbf{MODEL}_{base}$	$\%_{base}$	$\textbf{MODEL}_{R0063}$	$\%_{R0063}$	$\textbf{MODEL}_{R0065}$	$\%_{R0065}$	$\textbf{MODEL}_{R0068}$	$\%_{R0068}$	$\textbf{MODEL}_{R0111}$	$\%_{R0111}$
Elimination	0	2269	31.5	2441	34.0	2217	30.8	2222	30.9	2273	31.6
	1	4929	68.5	4742	66.0	4982	69.2	4976	69.1	4927	68.4
$p < 0.0001$	all	7198	100.0	7183	100.0	7199	100.0	7198	100.0	7200	100.0
Resurgence	0	3375	46.9	3398	47.3	3863	53.7	3529	49.0	3442	47.8
	1	3823	53.1	3785	52.7	3336	46.3	3669	51.0	3758	52.2
$p < 0.0001$	all	7198	100.0	7183	100.0	7199	100.0	7198	100.0	7200	100.0

**Variable**	**Levels**	$\textbf{MODEL}_{R0115}$	$\%_{R0115}$	$\textbf{MODEL}_{R0121}$	$\%_{R0121}$	$\textbf{MODEL}_{R0125}$	$\%_{R0125}$	$\textbf{MODEL}_{R0131}$	$\%_{R0131}$	$\textbf{MODEL}_{R0132}$	$\%_{R0132}$
Elimination	0	2104	29.2	2247	31.2	2081	28.9	2183	30.3	2149	29.9
	1	5096	70.8	4953	68.8	5118	71.1	5017	69.7	5051	70.2
$p < 0.0001$	all	7200	100.0	7200	100.0	7199	100.0	7200	100.0	7200	100.0
Resurgence	0	3715	51.6	3338	46.4	3174	44.1	3182	44.2	2939	40.8
	1	3485	48.4	3862	53.6	4025	55.9	4018	55.8	4261	59.2
$p < 0.0001$	all	7200	100.0	7200	100.0	7199	100.0	7200	100.0	7200	100.0

**Variable**	**Levels**	$\textbf{MODEL}_{R0133}$	$\%_{R0133}$	$\textbf{MODEL}_{R0670}$	$\%_{R0670}$	$\textbf{MODEL}_{R0674}$	$\%_{R0674}$	$\textbf{MODEL}_{R0678}$	$\%_{R0678}$	$\textbf{MODEL}_{all}$	$\%_{all}$
Elimination	0	2159	30.0	2207	30.6	2338	32.5	2342	32.5	31232	31.0
	1	5041	70.0	4993	69.3	4861	67.5	4858	67.5	69544	69.0
$p < 0.0001$	all	7200	100.0	7200	100.0	7199	100.0	7200	100.0	100776	100.0
Resurgence	0	3229	44.9	3323	46.1	2714	37.7	2572	35.7	45793	45.4
	1	3971	55.1	3877	53.9	4485	62.3	4628	64.3	54983	54.6
$p < 0.0001$	all	7200	100.0	7200	100.0	7199	100.0	7200	100.0	100776	100.0

Table S1: Simulation outputs for elimination and resurgence in terms of model variant for the African setting. Here column labeled **MODEL** shows the number of scenarios and the column labelled % shows the percentage of scenarios. The subscript denotes the model variant.

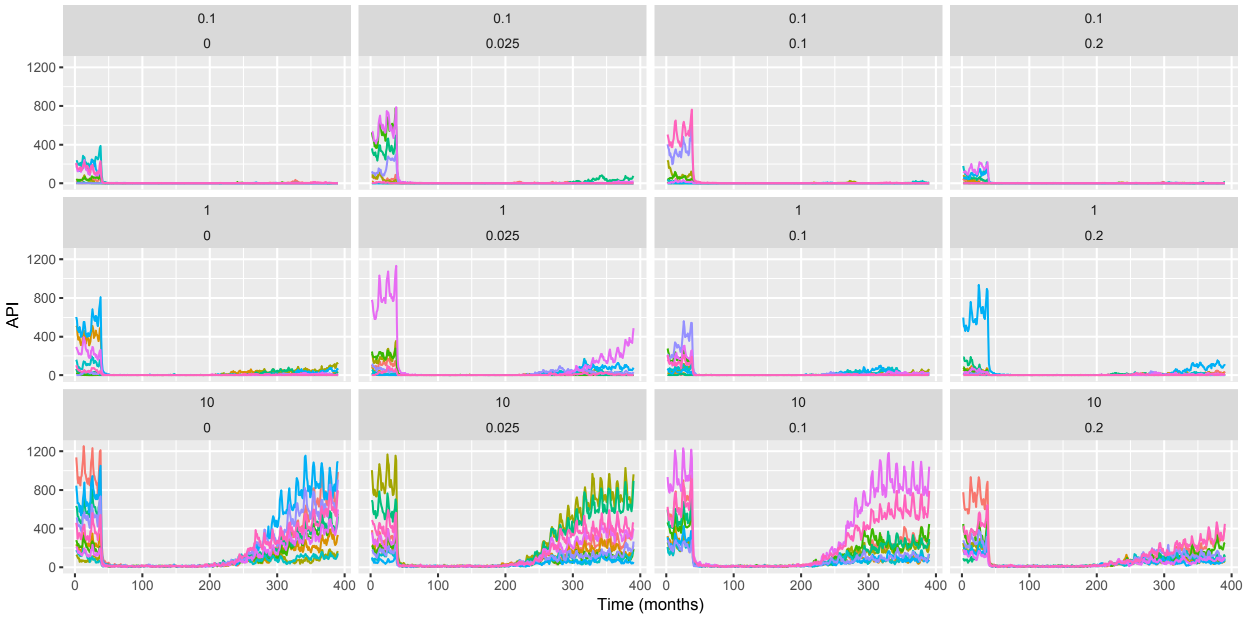

Figure S3: OpenMalaria simulation results for API per 1,000 per annum (Western Pacific scenario) with an annual pre-intervention entomological inoculation rate (EIR) of 0.1 infectious bites per adult per year, case management coverage of 80%, and LLIN coverage of 80% during the period of vector control implementation. API is the annual parasite incidence computed at each time step and the x-axis is in months. Each chart shows simulations results for varied levels of IIR and active surveillance mass screen and treat (MSAT) coverage). These values are shown just above each chart in the form: IIR per thousand per year, proportion of population tested per quarter. Colours of lines within the chart represent simulation runs with different random seeds (thus capturing stochastic uncertainty). Long lasting insecticidal nets (LLINs) are distributed at months 36, 72, 108, 144. Increased active surveillance starts immediately coincident with the last distribution of LLINs.

3.2 Western Pacific Setting

Simulation outputs allowed for the calculation of the time course of API, ABER and force of infection (FOI) for the Western Pacific setting. The results for API of sample simulations are shown in Figure S3. Figure S4 shows FOI for a subset of relevant simulation outputs. Results of a subset of simulations for ABER are shown in Figure S5.

Overall, there were 100,799 successfully completed simulations (only one simulation run failed to complete). Descriptive results are shown here in Tables S2–S8 for elimination and resurgence by various input parameters. Most simulations (65%) resulted in “elimination” during vector control roll-out. However, a similar fraction (61%) of simulations showed resurgence after vector control withdrawal (Table S2). When results for elimination and resurgence were examined in bivariate analysis for background characteristics of simulation, elimination during the vector-control period was associated with level of vector control coverage achieved (Table S3), case management coverage (Table S4), baseline pre-intervention entomological inoculation rate (EIR) (Table S5), infection importation rate (Table S6), and

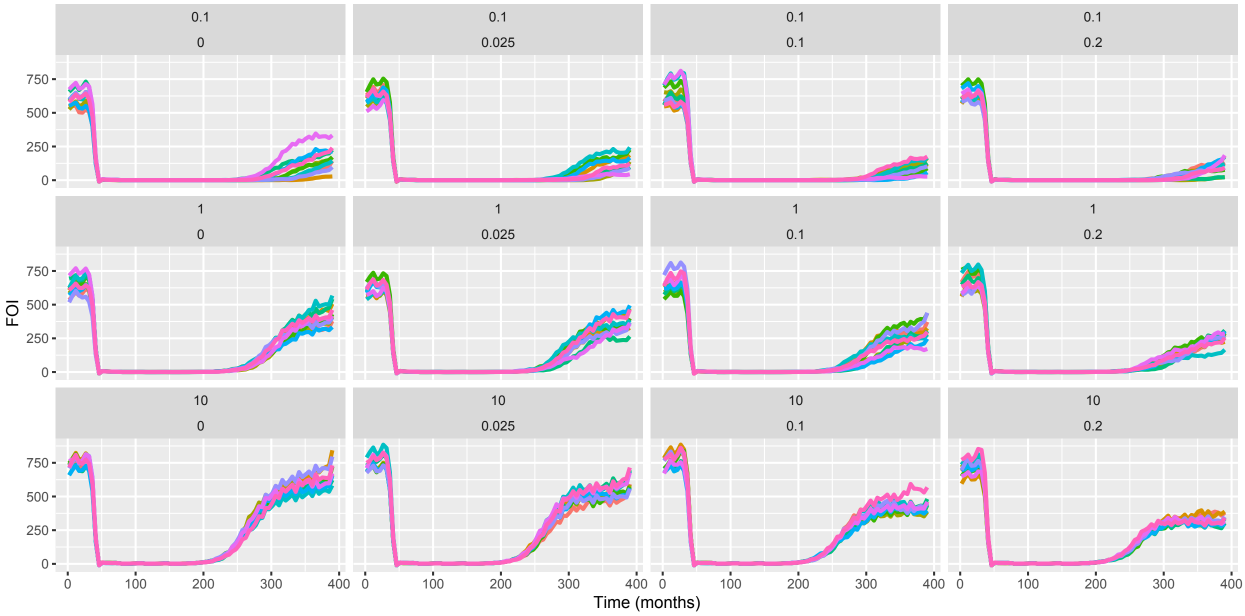

Figure S4: OpenMalaria simulation results for FOI per 1,000 per annum (Western Pacific scenario) with an annual pre-intervention EIR of 1 infectious bite per adult per year, case management coverage of 80%, and LLIN coverage of 80% during the period of vector control implementation. FOI is the number of new infections (including super-infections) over the previous month (normalised to units of infections per 1,000 people per year) and the x-axis is in months. Each chart shows simulations results for varied levels of IIR and active surveillance (through quarterly MSAT coverage). These values are shown just above each chart in the form: IIR per thousand per year, proportion of population tested per quarter. Colours of lines within the chart represent simulation runs with different random seeds (thus capturing stochastic uncertainty). LLINs are distributed at months 36, 72, 108, 144. Increased active surveillance starts immediately coincident with the last distribution of LLINs.

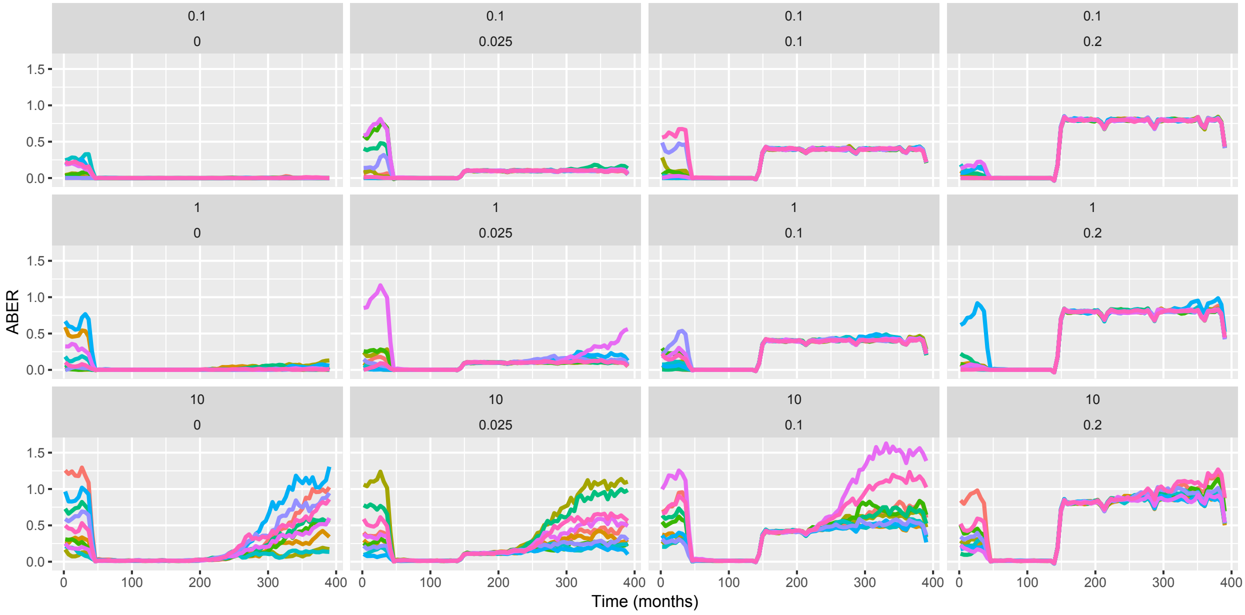

Figure S5: OpenMalaria simulation results for ABER (Western Pacific scenario) with an annual pre-intervention EIR of 0.1 infectious bites per adult per year, case management coverage of 80%, and LLIN coverage of 80% during the period of vector control implementation. ABER represents the number of diagnostic tests used per person over the previous month (smoothed to remove the visual effects of widely varying ABER between time periods with quarterly MSAT surveys and normalised to units of tests per person per year) and the x-axis is in months. Each chart shows simulations results for varied levels of IIR and active surveillance (through quarterly MSAT coverage). These values are shown just above each chart in the form: IIR per thousand per year, proportion of population tested per quarter. Colours of lines within the chart represent simulation runs with different random seeds (thus capturing stochastic uncertainty). LLINs are distributed at months 36, 72, 108, 144. Increased active surveillance starts immediately coincident with the last distribution of LLINs.

model variant (Table S8). Resurgence during the post-vector control period was associated with the level of vector control coverage achieved, case management coverage, baseline pre-intervention EIR, infection importation rate, active surveillance coverage, and model variant (Tables S3–S8).

Table S2 shows the proportion of simulations which resulted in elimination and resurgence. In the majority of simulations (65%) the level of malaria transmission during vector control deployment met the criteria for elimination during vector control deployment. The majority of simulations (61%) also resulted in a resurgence in the post vector control period. Of the scenarios where malaria was eliminated, close to half showed resurgence (44%). Due to stochastic variation, there was a small proportion (6%) of scenarios that showed no resurgence even though malaria had not been eliminated.

**Variable**	**Levels**	$\mathbf{ELIM}_0$	$\%_0$	$\mathbf{ELIM}_1$	$\%_1$	$\mathbf{ELIM}_{\mathrm{all}}$	$\%_{\mathrm{all}}$
Elimination	0	35468	100.0	0	0.0	35468	35.2
	1	0	0.0	65331	100.0	65331	64.8
$p < 0.0001$	all	35468	100.0	65331	100.0	100799	100.0
Resurgence	0	1987	5.6	36945	56.5	38932	38.6
	1	33481	94.4	28386	43.5	61867	61.4
$p < 0.0001$	all	35468	100.0	65331	100.0	100799	100.0

Table S2: Simulation outputs for elimination and resurgence: here column labelled **ELIM** shows the number of scenarios and the column labelled % shows the percentage of scenarios. The subscript 0 denotes scenarios where elimination did not occur; 1 denotes scenarios where elimination occurred; and all denotes all scenarios.

Increasing coverage of LLINs during vector control deployment was associated with increased probabilities of elimination and as well as reduced probabilities of resurgence (Table S3).

**Variable**	**Levels**	$\mathbf{ITN}_0$	$\%_0$	$\mathbf{ITN}_{0.2}$	$\%_{0.2}$	$\mathbf{ITN}_{0.5}$	$\%_{0.5}$	$\mathbf{ITN}_{0.8}$	$\%_{0.8}$	$\mathbf{ITN}_{\mathrm{all}}$	$\%_{\mathrm{all}}$
Elimination	0	24157	95.9	9597	38.1	1683	6.7	31	0.1	35468	35.2
	1	1043	4.1	15603	61.9	23516	93.3	25169	99.9	65331	64.8
$p < 0.0001$	all	25200	100.0	25200	100.0	25199	100.0	25200	100.0	100799	100.0
Resurgence	0	1233	4.9	9606	38.1	13252	52.6	14841	58.9	38932	38.6
	1	23967	95.1	15594	61.9	11947	47.4	10359	41.1	61867	61.4
$p < 0.0001$	all	25200	100.0	25200	100.0	25199	100.0	25200	100.0	100799	100.0

Table S3: Simulation outputs for elimination and resurgence in terms of LLIN coverage during vector control (Solomon Islands): here column labelled **ITN** shows the number of scenarios and the column labelled % shows the percentage of scenarios. The subscript denotes the proportion of the population sleeping under LLINs during the vector control period.

Changes in the level of case management coverage were associated with differences in both the probability of elimination and resurgence (Table S4).

**Variable**	**Levels**	$\mathbf{CM}_{0.2}$	$\%_{0.2}$	$\mathbf{CM}_{0.5}$	$\%_{0.5}$	$\mathbf{CM}_{0.8}$	$\%_{0.8}$	$\mathbf{CM}_{\text{all}}$	$\%_{\text{all}}$
Elimination	0	13358	39.8	11582	34.5	10528	31.3	35468	35.2
	1	20242	60.2	22017	65.5	23072	68.7	65331	64.8
$p < 0.0001$	all	33600	100.0	33599	100.0	33600	100.0	100799	100.0
Resurgence	0	9818	29.2	13348	39.7	15766	46.9	38932	38.6
	1	23782	70.8	20251	60.3	17834	53.1	61867	61.4
$p < 0.0001$	all	33600	100.0	33599	100.0	33600	100.0	100799	100.0

Table S4: Simulation outputs for elimination and resurgence in terms of case management coverage (Solomon Islands): here column labelled **CM** shows the number of scenarios and the column labelled % shows the percentage of scenarios. The subscript denotes the proportion of cases of malaria receiving effective treatment.

Pre-intervention EIR was strongly associated with probabilities of both elimination and resurgence. These associations showed trends in the expected directions with elimination much less likely to occur at higher baseline pre-intervention EIRs and resurgence much more likely to occur at higher pre-intervention EIRs (Table S5).

**Variable**	**Levels**	$\mathbf{EIR}_{0.1}$	$\%_{0.1}$	$\mathbf{EIR}_{0.5}$	$\%_{0.5}$	$\mathbf{EIR}_{1}$	$\%_{1}$	$\mathbf{EIR}_{2}$	$\%_{2}$	$\mathbf{EIR}_{5}$	$\%_{5}$	$\mathbf{EIR}_{\text{all}}$	$\%_{\text{all}}$
Elimination	0	4161	20.6	5074	25.2	5961	29.6	8502	42.2	11770	58.4	35468	35.2
	1	15999	79.4	15086	74.8	14198	70.4	11658	57.8	8390	41.6	65331	64.8
$p < 0.0001$	all	20160	100.0	20160	100.0	20159	100.0	20160	100.0	20160	100.0	100799	100.0
Resurgence	0	13878	68.8	10161	50.4	7872	39.0	5328	26.4	1693	8.4	38932	38.6
	1	6282	31.2	9999	49.6	12287	61.0	14832	73.6	18467	91.6	61867	61.4
$p < 0.0001$	all	20160	100.0	20160	100.0	20159	100.0	20160	100.0	20160	100.0	100799	100.0

Table S5: Simulation outputs for elimination and resurgence in terms of baseline pre-intervention EIR (Solomon Islands): here column labelled **EIR** shows the number of scenarios and the column labelled % shows the percentage of scenarios. The subscript denotes the pre-intervention EIR in units of infectious bites per adult per year.

IIR was associated with the probability of resurgence and elimination (Table S6).

**Variable**	**Levels**	$\mathbf{IIR}_{0.1}$	$\%_{0.1}$	$\mathbf{IIR}_{1}$	$\%_{1}$	$\mathbf{IIR}_{10}$	$\%_{10}$	$\mathbf{IIR}_{all}$	$\%_{all}$
Elimination	0	12420	37.0	11966	35.6	11082	33.0	35468	35.2
	1	21180	63.0	21634	64.4	22517	67.0	65331	64.8
$p < 0.0001$	all	33600	100.0	33600	100.0	33599	100.0	100799	100.0
Resurgence	0	19693	58.6	11941	35.5	7298	21.7	38932	38.6
	1	13907	41.4	21659	64.5	26301	78.3	61867	61.4
$p < 0.0001$	all	33600	100.0	33600	100.0	33599	100.0	100799	100.0

Table S6: Simulation outputs for elimination and resurgence in terms of infection importation rate per 1,000 per annum (Solomon Islands): here column labelled **IIR** shows the number of scenarios and the column labelled % shows the percentage of scenarios. The subscript denotes the Infection Importation Rate per 1,000 person-years.

Changes in active surveillance coverage across the range tested was not related to the probability of elimination but was for resurgence. Since active surveillance was not deployed during the period of vector control in these simulations the lack of any association with elimination during vector control is expected (Table S7).

**Variable**	**Levels**	$\mathbf{AS}_{0}$	$\%_{0}$	$\mathbf{AS}_{0.025}$	$\%_{0.025}$	$\mathbf{AS}_{0.1}$	$\%_{0.1}$	$\mathbf{AS}_{0.2}$	$\%_{0.2}$	$\mathbf{AS}_{all}$	$\%_{all}$
Elimination	0	8873	35.2	8906	35.3	8839	35.1	8850	35.1	35468	35.2
	1	16327	64.8	16293	64.7	16361	64.9	16350	64.9	65331	64.8
$p = 0.93$	all	25200	100.0	25199	100.0	25200	100.0	25200	100.0	100799	100.0
Resurgence	0	8570	34.0	8936	35.5	10067	40.0	11359	45.1	38932	38.6
	1	16630	66.0	16263	64.5	15133	60.0	13841	54.9	61867	61.4
$p < 0.0001$	all	25200	100.0	25199	100.0	25200	100.0	25200	100.0	100799	100.0

Table S7: Simulation outputs for elimination and resurgence in terms of active surveillance coverage (Solomon Islands): here column labelled **AS** shows the number of scenarios and the column labelled % shows the percentage of scenarios. The subscript denotes the proportion of the population covered by active surveillance in each quarter.

Variable	Levels	$MODEL_{base}$	$\%_{base}$	$MODEL_{R0063}$	$\%_{R0063}$	$MODEL_{R0065}$	$\%_{R0065}$	$MODEL_{R0068}$	$\%_{R0068}$	$MODEL_{R0111}$	$\%_{R0111}$
Elimination	0	2626	36.5	2889	40.1	2517	35.0	2495	34.6	2617	36.4
	1	4574	63.5	4311	59.9	4683	65.0	4705	65.3	4583	63.6
$p < 0.0001$	all	7200	100.0	7200	100.0	7200	100.0	7200	100.0	7200	100.0
Resurgence	0	2941	40.9	3083	42.8	3439	47.8	3138	43.6	2961	41.1
	1	4259	59.1	4117	57.2	3761	52.2	4062	56.4	4239	58.9
$p < 0.0001$	all	7200	100.0	7200	100.0	7200	100.0	7200	100.0	7200	100.0

Variable	Levels	$MODEL_{R0115}$	$\%_{R0115}$	$MODEL_{R0121}$	$\%_{R0121}$	$MODEL_{R0125}$	$\%_{R0125}$	$MODEL_{R0131}$	$\%_{R0131}$	$MODEL_{R0132}$	$\%_{R0132}$
Elimination	0	2359	32.8	2577	35.8	2358	32.8	2444	33.9	2375	33.0
	1	4841	67.2	4623	64.2	4841	67.2	4756	66.1	4825	67.0
$p < 0.0001$	all	7200	100.0	7200	100.0	7199	100.0	7200	100.0	7200	100.0
Resurgence	0	3074	42.7	2889	40.1	2552	35.5	2604	36.2	2380	33.1
	1	4126	57.3	4311	59.9	4647	64.5	4596	63.8	4820	66.9
$p < 0.0001$	all	7200	100.0	7200	100.0	7199	100.0	7200	100.0	7200	100.0

Variable	Levels	$MODEL_{R0133}$	$\%_{R0133}$	$MODEL_{R0670}$	$\%_{R0670}$	$MODEL_{R0674}$	$\%_{R0674}$	$MODEL_{R0678}$	$\%_{R0678}$	$MODEL_{all}$	$\%_{all}$
Elimination	0	2335	32.4	2500	34.7	2680	37.2	2696	37.4	35468	35.2
	1	4865	67.6	4700	65.3	4520	62.8	4504	62.6	65331	64.8
$p < 0.0001$	all	7200	100.0	7200	100.0	7200	100.0	7200	100.0	100799	100.0
Resurgence	0	2586	35.9	2801	38.9	2289	31.8	2195	30.5	38932	38.6
	1	4614	64.1	4399	61.1	4911	68.2	5005	69.5	61867	61.4
$p < 0.0001$	all	7200	100.0	7200	100.0	7200	100.0	7200	100.0	100799	100.0

Table S8: Simulation outputs for elimination and resurgence in terms of model variant for the Western Pacific setting. Here the column labeled **MODEL** shows the number of scenarios and the column labeled % shows the percentage of scenarios. The subscript denotes the model variant.

Model variant was also associated with the probability of resurgence and elimination (Table S8).

4 Regression Results: Western Pacific Setting

We applied logistic regression using input parameters and malaria outcomes during vector control interventions of each simulation as predictors; and the probability of resurgence post withdrawal of vector control as the outcome (Table S9).

These results indicate that most parameters which were important in bivariate analysis retained important predictive value for the probability of a resurgence in multivariate analysis. Overall model results reinforce the importance of pre-intervention EIR, case management coverage, active surveillance coverage, infection importation and the level of control success during vector control deployment as major driving factors in predicting the probability of resurgence after withdrawal.

These logistic regression model results can be used to summarise the predicted probability of a resurgence occurring with varying levels of input parameters. Figure S6 shows the predicted probability of resurgence at varying levels of API, IIR, EIR and active surveillance coverage for the base model variant.

The predicted probability of resurgence is generally high for most parameter combinations and only falls below 0.25 for a set of simulations in which input EIR was less than 1, IIR was 1 per 1,000 per year, mean API during vector control deployment was below 25 per person per year and there was some level of active surveillance.

We also analyzed the time to resurgence by fitting a Cox-proportional hazard model to these time to event outcomes, assuming that the simulations in which no resurgence occurred were right censored. The results of this regres-

Table S9: Logistic regression of input model parameters on resurgence (Solomon Islands)

	Dependent variable:	
	Resurgence	95% C.I.
Mean API During VC (per 1000)	1.082***	(1.077, 1.086)
Case Management Cov.	0.038***	(0.035, 0.042)
EIR	2.828***	(2.774, 2.883)
(10x) Active Surv. Cov.	0.559***	(0.545, 0.574)
0.2 ITN	0.262***	(0.233, 0.296)
0.5 ITN	0.140***	(0.123, 0.160)
0.8 ITN	0.093***	(0.081, 0.106)
IIR 1	9.880***	(9.331, 10.465)
IIR 10	15.476***	(14.375, 16.663)
R0063	0.702***	(0.631, 0.781)
R0065	0.496***	(0.446, 0.552)
R0068	0.783***	(0.704, 0.870)
R0111	0.944	(0.849, 1.049)
R0115	0.852***	(0.766, 0.947)
R0121	1.061	(0.955, 1.179)
R0125	1.722***	(1.550, 1.913)
R0131	1.612***	(1.451, 1.791)
R0132	2.163***	(1.946, 2.405)
R0133	1.656***	(1.491, 1.841)
R0670	1.192***	(1.073, 1.324)
R0674	2.381***	(2.141, 2.648)
R0678	2.751***	(2.473, 3.060)
Constant	1.027	(0.889, 1.186)
Observations	100,799	
Log Likelihood	−29,689.990	
Akaike Inf. Crit.	59,425.980	
Note:	*p<0.1; **p<0.05; ***p<0.01	

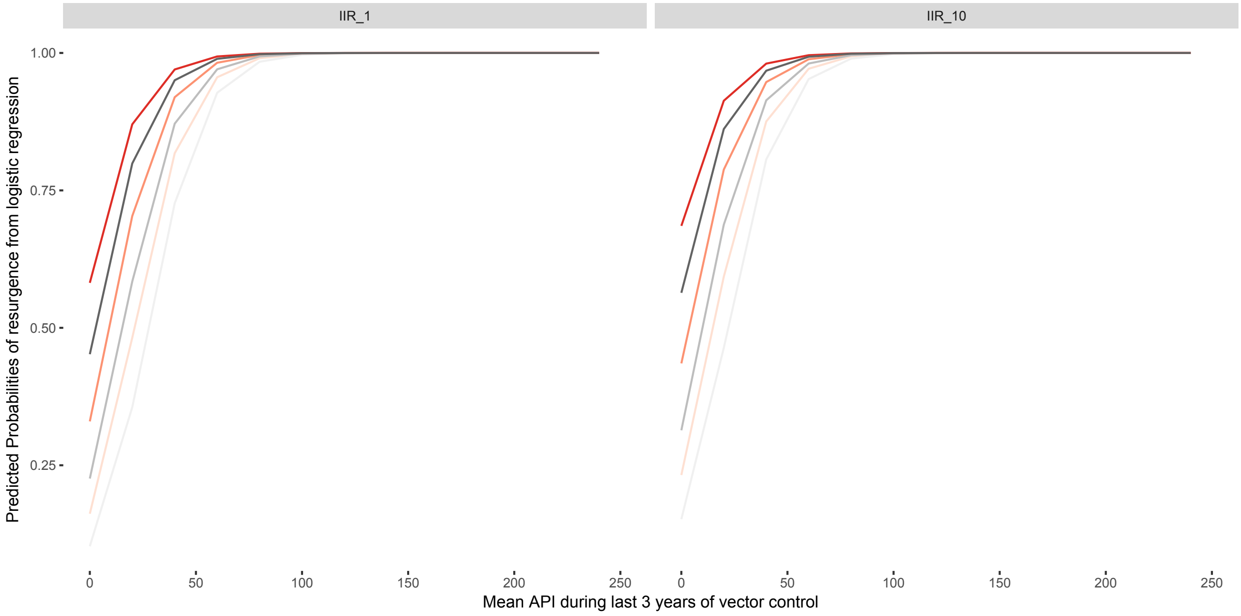

Figure S6: Predicted probabilities of resurgence based on regression results in Table S9 (Solomon Islands scenario) plotted against the mean API during the last three years of vector control (years **9–12**). Darker lines represent increasing EIR $\{0.1, 1, 2\}$, while grey lines represent active surveillance coverage of 1% per quarter and red lines represent active surveillance coverage of 10% per quarter. The plot on the left is for an IIR of 1 imported infection per 1000 people per year while the plot on the right is for an IIR of 10 imported infections per 1000 people per year. All slopes here are for LLIN coverage of 80%, case management coverage of 50% and the base model variant.

sion are summarised in Table S10.

Table S10: Cox model regression of input model parameters on time to resurgence

	Dependent variable:	
	Time till resurgence	95% C.I.
Mean API During VC (per 1000)	1.032***	(1.030, 1.033)
Case Management Cov.	0.508***	(0.491, 0.525)
EIR	1.271***	(1.265, 1.278)
(10x) Active Surv. Cov.	0.890***	(0.881, 0.899)
ITN coverage	0.288***	(0.278, 0.298)
IIR	1.083***	(1.081, 1.086)
R0063	0.745***	(0.715, 0.776)
R0065	0.775***	(0.745, 0.807)
R0068	0.898***	(0.863, 0.935)
R0111	0.982	(0.943, 1.023)
R0115	0.947***	(0.910, 0.986)
R0121	1.025	(0.984, 1.067)
R0125	1.074***	(1.032, 1.118)
R0131	1.093***	(1.050, 1.137)
R0132	1.112***	(1.068, 1.157)
R0133	1.085***	(1.043, 1.130)
R0670	1.013	(0.973, 1.055)
R0674	1.101***	(1.057, 1.146)
R0678	1.160***	(1.113, 1.208)
Observations	67,916	
R^2	0.292	
Max. Possible R^2	1.000	
Log Likelihood	−665,758.000	
Wald Test	25,192.550*** (df = 19)	
LR Test	23,458.420*** (df = 19)	
Score (Logrank) Test	27,399.380*** (df = 19)	
Note:	*p<0.1; **p<0.05; ***p<0.01	

Similarly we used the severity of resurgence for the outcome in the linear regression as previously defined for the analysis of the Kenya simulations (Table S11).

5 Sensitivity Analysis of Resurgence and Elimination Threshold

In order to examine the sensitivity of the results and recommendations to our choice of threshold for elimination and resurgence, we varied this threshold from the level used in the main analysis in the manuscript. In that analysis, elimination during the vector control period (between survey years **3** and **12**) was defined as occurring when the number of new infections in any one year was less than 3 times the 97.5 percentile of the Poisson distribution of the expected number of imported infections (in one year), as defined in a previous publication [1]. Similarly, we defined

Table S11: Linear regression of input model parameters on severity of resurgence (Solomon Islands)

	Dependent variable:	
	Severity	95% C.I.
Case Management Coverage	−20.783***	(−22.702, −18.865)
EIR	28.951***	(28.684, 29.219)
(10x) Active Surv. Cov.	−27.961***	(−28.565, −27.356)
0.2 ITN	125.722***	(124.392, 127.051)
0.5 ITN	114.727***	(113.397, 116.056)
0.8 ITN	103.999***	(102.670, 105.329)
IIR 1	17.723***	(16.572, 18.874)
IIR 10	49.973***	(48.821, 51.124)
R0063	−6.214***	(−8.701, −3.727)
R0065	−9.144***	(−11.631, −6.657)
R0068	−13.016***	(−15.503, −10.529)
R0111	−0.821	(−3.308, 1.666)
R0115	−5.535***	(−8.022, −3.048)
R0121	1.726	(−0.761, 4.213)
R0125	7.098***	(4.611, 9.585)
R0131	5.589***	(3.102, 8.076)
R0132	12.841***	(10.354, 15.328)
R0133	6.662***	(4.175, 9.150)
R0670	2.718**	(0.231, 5.205)
R0674	23.621***	(21.134, 26.108)
R0678	23.766***	(21.279, 26.253)
Constant	−101.468***	(−103.828, −99.108)
Observations	100,799	
R^2	0.515	
Adjusted R^2	0.515	
Residual Std. Error	76.141 (df = 100777)	
F Statistic	5,102.937*** (df = 21; 100777)	
Note:	*p<0.1; **p<0.05; ***p<0.01	

resurgence in the post-vector control period (between survey years **12** and **32**) as occurring when the number of new infections in any one year was greater than 3 times the 97.5 percentile of the Poisson distribution of the expected number of imported infections (in one year). We note here that the definition of resurgence is independent of the definition of elimination, so resurgence could occur in a scenario regardless of whether or not elimination occurred in that scenario. Additionally, because the observation period post vector control was purposefully set to be longer than the vector control period, there is more opportunity to observe resurgence than elimination due to stochastic variation.

For the purpose of sensitivity analysis we reduced or increased these thresholds to 2 or 4 times the 97.5 percentile of the Poisson distribution of the expected number of imported infections (in one year). This lower definition is consistent with the minimum number of infections at required to determine with statistical confidence that the number of cases during the post vector control exceeded the number of local cases required for conclusive proof of endemic transmission, while the higher threshold would be effectively double this number. The threshold used in our main analysis falls directly between these two.

We applied these new thresholds and conducted an analysis of the resulting outcomes using the same methods as for the main analysis, including descriptive analysis and logistic regression for both the Kenya scenario and the Solomon Islands scenario. Descriptive results on elimination and resurgence are shown in Tables S12, S13, S14, & S15. As expected in both the African Parameterizations and the Solomon Islands parameterization, reducing the threshold for resurgence and elimination leads to larger fractions of simulations experiencing resurgence, in this parameterization, resurgence is essentially coincident with re-establishment of endemic transmission after elimination. In both parameterizations, the models also indicate that raising the threshold for resurgence would limit the numbers of resurgences identified, however, in all cases a majority of simulations still resulted in resurgence.

**Variable**	**Levels**	$\mathbf{Elim}_0$	$\%_0$	$\mathbf{Elim}_1$	$\%_1$	$\mathbf{Elim}_{\text{all}}$	$\%_{\text{all}}$
Elimination.low	0	32550	100.0	0	0.0	32550	32.3
	1	0	0.0	68226	100.0	68226	67.7
$p < 0.0001$	all	32550	100.0	68226	100.0	100776	100.0
Resurgence.low	0	1575	4.8	37129	54.4	38704	38.4
	1	30975	95.2	31097	45.6	62072	61.6
$p < 0.0001$	all	32550	100.0	68226	100.0	100776	100.0

Table S12: Simulation outputs for elimination and resurgence with low threshold for the African setting: Here column labelled **Elim** shows the number of scenarios and the column labelled % shows the percentage of scenarios. The subscript 0 denotes scenarios where elimination did not occur; 1 denotes scenarios where elimination occurred; and all denotes all scenarios.

**Variable**	**Levels**	$\mathbf{Elim}_0$	$\%_0$	$\mathbf{Elim}_1$	$\%_1$	$\mathbf{Elim}_{\text{all}}$	$\%_{\text{all}}$
Elimination.high	0	30383	100.0	0	0.0	30383	30.1

	1	0	0.0	70393	100.0	70393	69.8
$p < 0.0001$	all	30383	100.0	70393	100.0	100776	100.0
Resurgence.high	0	1615	5.3	48367	68.7	49982	49.6
	1	28768	94.7	22026	31.3	50794	50.4
$p < 0.0001$	all	30383	100.0	70393	100.0	100776	100.0

Table S13: Simulation outputs for elimination and resurgence with high threshold for the African setting: Here column labelled **Elim** shows the number of scenarios and the column labelled % shows the percentage of scenarios. The subscript 0 denotes scenarios where elimination did not occur; 1 denotes scenarios where elimination occurred; and all denotes all scenarios.

**Variable**	**Levels**	$\mathbf{Elim}_0$	$\%_0$	$\mathbf{Elim}_1$	$\%_1$	$\mathbf{Elim}_{\text{all}}$	$\%_{\text{all}}$
Elimination.low	0	37856	100.0	0	0.0	37856	37.6
	1	0	0.0	62943	100.0	62943	62.4
$p < 0.0001$	all	37856	100.0	62943	100.0	100799	100.0
Resurgence.low	0	2175	5.8	29736	47.2	31911	31.7
	1	35681	94.2	33207	52.8	68888	68.3
$p < 0.0001$	all	37856	100.0	62943	100.0	100799	100.0

Table S14: Simulation outputs for elimination and resurgence with low threshold for the Western Pacific setting: Here column labelled **Elim** shows the number of scenarios and the column labelled % shows the percentage of scenarios. The subscript 0 denotes scenarios where elimination did not occur; 1 denotes scenarios where elimination occurred; and all denotes all scenarios.

**Variable**	**Levels**	$\mathbf{Elim}_0$	$\%_0$	$\mathbf{Elim}_1$	$\%_1$	$\mathbf{Elim}_{\text{all}}$	$\%_{\text{all}}$
Elimination.high	0	34262	100.0	0	0.0	34262	34.0
	1	0	0.0	66537	100.0	66537	66.0
$p < 0.0001$	all	34262	100.0	66537	100.0	100799	100.0
Resurgence.high	0	1957	5.7	41663	62.6	43620	43.3
	1	32305	94.3	24874	37.4	57179	56.7
$p < 0.0001$	all	34262	100.0	66537	100.0	100799	100.0

Table S15: Simulation outputs for elimination and resurgence with high threshold for the Western Pacific setting: Here column labelled **Elim** shows the number of scenarios and the column labelled % shows the percentage of scenarios. The subscript 0 denotes scenarios where elimination did not occur; 1 denotes scenarios where elimination occurred; and all denotes all scenarios.

In order to determine if the threshold for elimination had a meaningful effect on the determinants and probabilities

of resurgence we also applied logistic regression to these results. The results of these four regressions are shown in Tables S16, S17, S18 & S19. Overall, raising or lowering the threshold for elimination or resurgence did not substantially affect the magnitude or direction of any regression coefficients used for predicting the probability of resurgence. Though the changes do produce noticeable shifts in the predicted probability of resurgence under different scenarios, indicating that the understanding the background of transmission and the history of vector control in an area are important to determining the probability of resurgence. Further, these results show that the definitions of resurgence are an important consideration in a full risk model.

Table S16: Logistic regression of input model parameters on resurgence with low threshold for the African setting

	Dependent variable:	
	Resurgence (low)	
Mean API During VC (per 1000)	1.110***	(1.103, 1.117)
Case Management Cov.	0.037***	(0.034, 0.040)
EIR	2.782***	(2.731, 2.835)
(10x) Active Surv. Cov.	0.614***	(0.598, 0.630)
0.2 ITN	0.204***	(0.182, 0.229)
0.5 ITN	0.088***	(0.078, 0.100)
0.8 ITN	0.054***	(0.047, 0.061)
IIR 1	11.307***	(10.673, 11.984)
IIR 10	22.447***	(20.666, 24.378)
R0063	0.715***	(0.643, 0.796)
R0065	0.465***	(0.418, 0.518)
R0068	0.827***	(0.744, 0.919)
R0111	0.841***	(0.757, 0.935)
R0115	0.780***	(0.702, 0.867)
R0121	1.035	(0.931, 1.150)
R0125	1.585***	(1.426, 1.762)
R0131	1.445***	(1.301, 1.606)
R0132	1.839***	(1.654, 2.046)
R0133	1.419***	(1.277, 1.578)
R0670	1.180***	(1.062, 1.311)
R0674	2.236***	(2.010, 2.488)
R0678	2.753***	(2.473, 3.065)
Constant	1.407***	(1.227, 1.612)
Observations	100,776	
Log Likelihood	−29,522.850	
Akaike Inf. Crit.	59,091.690	
Note:	*p<0.1; **p<0.05; ***p<0.01	

References

[1] Crowell, V., Hardy, D., Briët, O., Chitnis, N., Maire, N., Smith, T.: Can we depend on case management to prevent

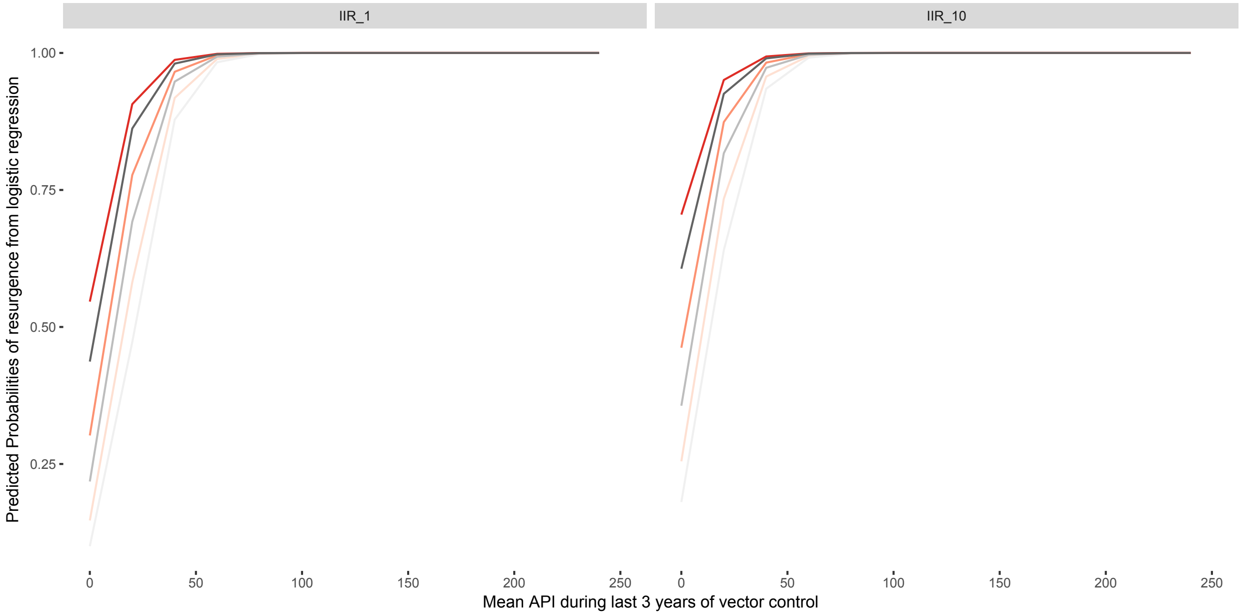

Figure S7: Predicted probabilities of resurgence (low threshold) based on regression results in Table S16 (African Scenario) plotted against the mean API during the last three years of vector control (years **9–12**). Darker lines represent increasing EIR {0.1, 1, 2}, while grey lines represent active surveillance coverage of 1% per quarter and red lines represent active surveillance coverage of 10% per quarter. The plot on the left is for an IIR of 1 imported infection per 1000 people per year while the plot on the right is for an IIR of 10 imported infections per 1000 people per year. All slopes here are for LLIN coverage of 80%, case management coverage of 50% and the base model variant.

Table S17: Logistic regression of input model parameters on resurgence with high threshold for the African setting

	Dependent variable:	
	Resurgence (high)	
Mean API During VC (per 1000)	1.050***	(1.047, 1.053)
Case Management Cov.	0.015***	(0.013, 0.016)
EIR	3.595***	(3.522, 3.670)
(10x) Active Surv. Cov.	0.573***	(0.556, 0.590)
0.2 ITN	0.135***	(0.121, 0.150)
0.5 ITN	0.056***	(0.050, 0.063)
0.8 ITN	0.033***	(0.029, 0.038)
IIR 1	9.096***	(8.538, 9.694)
IIR 10	12.915***	(12.042, 13.856)
R0063	0.903*	(0.804, 1.013)
R0065	0.469***	(0.416, 0.529)
R0068	0.793***	(0.706, 0.890)
R0111	0.876**	(0.780, 0.983)
R0115	0.554***	(0.493, 0.623)
R0121	1.014	(0.904, 1.137)
R0125	1.258***	(1.123, 1.408)
R0131	1.242***	(1.109, 1.391)
R0132	1.758***	(1.572, 1.967)
R0133	1.097	(0.979, 1.230)
R0670	0.981	(0.875, 1.100)
R0674	2.641***	(2.362, 2.954)
R0678	3.280***	(2.934, 3.668)
Constant	1.332***	(1.160, 1.530)
Observations	100,776	
Log Likelihood	−26,411.730	
Akaike Inf. Crit.	52,869.460	
Note:	*p<0.1; **p<0.05; ***p<0.01	

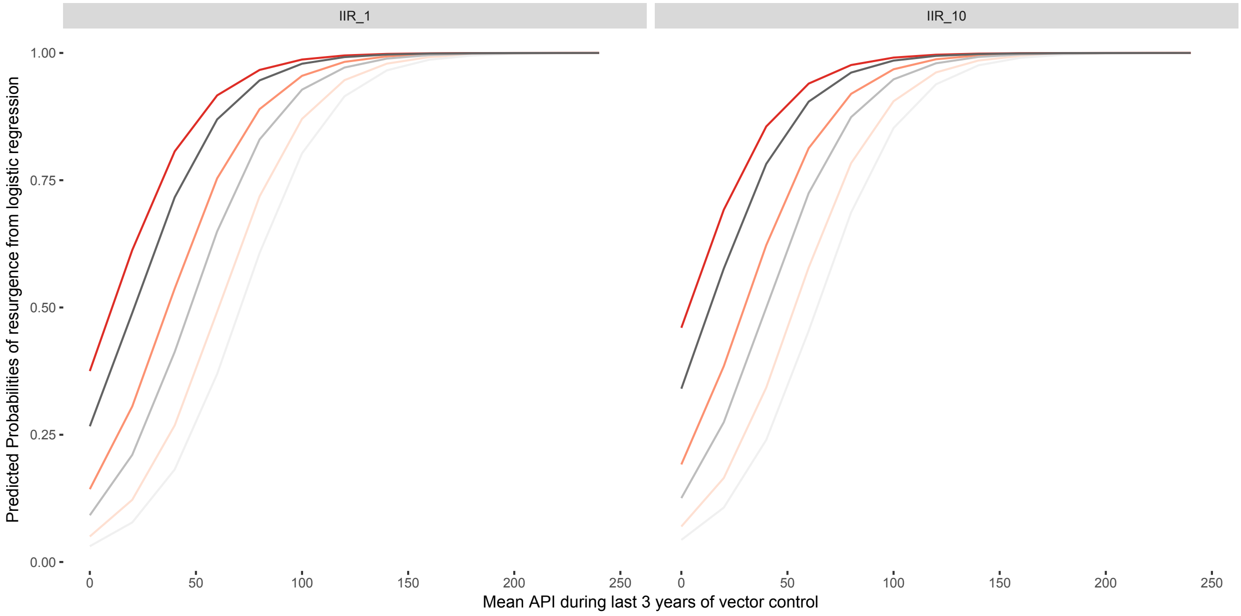

Figure S8: Predicted probabilities of resurgence based on regression results in Table S17 (African Scenario) plotted against the mean API during the last three years of vector control (years **9–12**). Darker lines represent increasing EIR {0.1, 1, 2}, while grey lines represent active surveillance coverage of 1% per quarter and red lines represent active surveillance coverage of 10% per quarter. The plot on the left is for an IIR of 1 imported infection per 1000 people per year while the plot on the right is for an IIR of 10 imported infections per 1000 people per year. All slopes here are for LLIN coverage of 80%, case management coverage of 50% and the base model variant.

Table S18: Logistic regression of input model parameters on resurgence with low threshold for the Western Pacific setting

	Dependent variable:	
	Resurgence (low)	
Mean API During VC (per 1000)	1.107***	(1.101, 1.114)
Case Management Cov.	0.053***	(0.048, 0.058)
EIR	2.395***	(2.351, 2.441)
(10x) Active Surv. Cov.	0.586***	(0.570, 0.602)
0.2 ITN	0.308***	(0.271, 0.349)
0.5 ITN	0.162***	(0.142, 0.185)
0.8 ITN	0.108***	(0.094, 0.124)
IIR 1	11.435***	(10.828, 12.081)
IIR 10	28.275***	(25.974, 30.780)
R0063	0.597***	(0.536, 0.665)
R0065	0.472***	(0.424, 0.525)
R0068	0.806***	(0.725, 0.897)
R0111	0.953	(0.856, 1.062)
R0115	1.028	(0.923, 1.144)
R0121	1.128**	(1.013, 1.257)
R0125	1.797***	(1.611, 2.004)
R0131	1.666***	(1.495, 1.858)
R0132	2.040***	(1.828, 2.277)
R0133	1.710***	(1.534, 1.907)
R0670	1.268***	(1.138, 1.413)
R0674	2.243***	(2.009, 2.505)
R0678	2.508***	(2.246, 2.802)
Constant	1.301***	(1.122, 1.510)
Observations	100,799	
Log Likelihood	−28,315.880	
Akaike Inf. Crit.	56,677.760	
Note:	*p<0.1; **p<0.05; ***p<0.01	

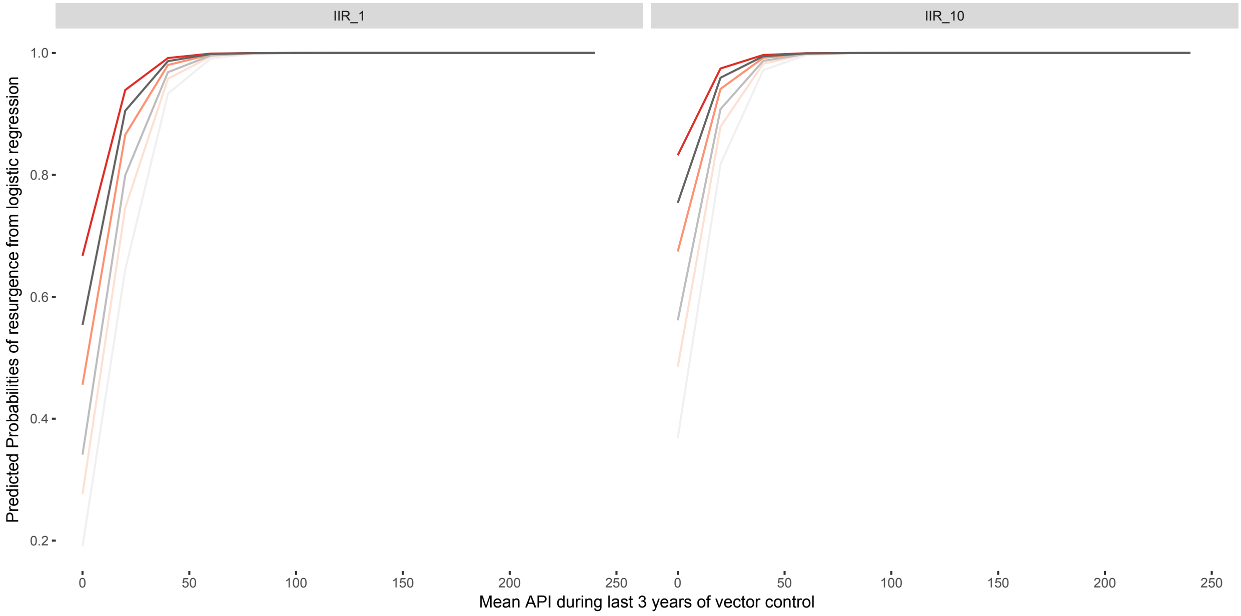

Figure S9: Predicted probabilities of resurgence based on regression results in Table S18 (Solomon Islands Scenario) plotted against the mean API during the last three years of vector control (years **9–12**). Darker lines represent increasing EIR {0.1, 1, 2}, while grey lines represent active surveillance coverage of 1% per quarter and red lines represent active surveillance coverage of 10% per quarter. The plot on the left is for an IIR of 1 imported infection per 1000 people per year while the plot on the right is for an IIR of 10 imported infections per 1000 people per year. All slopes here are for LLIN coverage of 80%, case management coverage of 50% and the base model variant.

Table S19: Logistic regression of input model parameters on resurgence with high threshold for the Western Pacific setting

	Dependent variable:	
	Resurgence (high)	
Mean API During VC (per 1000)	1.057^{***}	(1.054, 1.060)
Case Management Cov.	0.027^{***}	(0.025, 0.030)
EIR	3.170^{***}	(3.108, 3.234)
(10x) Active Surv. Cov.	0.550^{***}	(0.535, 0.565)
0.2 ITN	0.234^{***}	(0.208, 0.262)
0.5 ITN	0.117^{***}	(0.103, 0.133)
0.8 ITN	0.077^{***}	(0.067, 0.087)
IIR 1	8.653^{***}	(8.160, 9.180)
IIR 10	12.059^{***}	(11.264, 12.913)
R0063	0.761^{***}	(0.682, 0.849)
R0065	0.479^{***}	(0.428, 0.535)
R0068	0.754^{***}	(0.676, 0.841)
R0111	0.848^{***}	(0.761, 0.946)
R0115	0.677^{***}	(0.607, 0.755)
R0121	1.026	(0.921, 1.143)
R0125	1.598^{***}	(1.436, 1.778)
R0131	1.452^{***}	(1.305, 1.615)
R0132	2.226^{***}	(2.001, 2.476)
R0133	1.496^{***}	(1.345, 1.665)
R0670	1.057	(0.949, 1.177)
R0674	2.641^{***}	(2.374, 2.940)
R0678	2.866^{***}	(2.576, 3.190)
Constant	1.052	(0.912, 1.213)
Observations	100,799	
Log Likelihood	−28,967.320	
Akaike Inf. Crit.	57,980.640	
Note:	*p<0.1; **p<0.05; ***p<0.01	

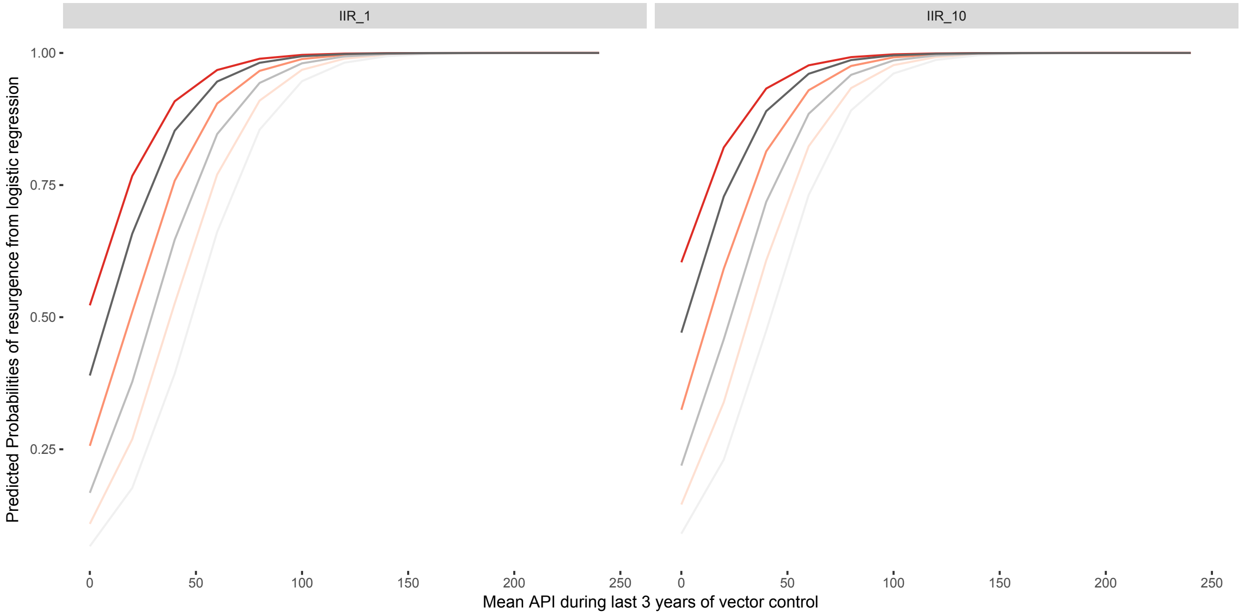

Figure S10: Predicted probabilities of resurgence (high threshold) based on regression results in Table S19 (Solomon Islands Scenario) plotted against the mean API during the last three years of vector control (years **9–12**). Darker lines represent increasing EIR {0.1, 1, 2}, while grey lines represent active surveillance coverage of 1% per quarter and red lines represent active surveillance coverage of 10% per quarter. The plot on the left is for an IIR of 1 imported infection per 1000 people per year while the plot on the right is for an IIR of 10 imported infections per 1000 people per year. All slopes here are for LLIN coverage of 80%, case management coverage of 50% and the base model variant.

re-establishment of *P. falciparum* malaria, after local interruption of transmission? Epidemics **4**(1), 1–8 (2012)
